# Single-cell T cell receptor sequencing of paired human atherosclerotic plaques and blood reveals autoimmune-like features of expanded effector T cells

**DOI:** 10.1038/s44161-022-00208-4

**Published:** 2023-01-30

**Authors:** Marie A. C. Depuydt, Frank H. Schaftenaar, Koen H. M. Prange, Arjan Boltjes, Esmeralda Hemme, Lucie Delfos, Jill de Mol, Maaike J. M. de Jong, Mireia N. A. Bernabé Kleijn, Judith A. H. M. Peeters, Lauren Goncalves, Anouk Wezel, Harm J. Smeets, Gert J. de Borst, Amanda C. Foks, Gerard Pasterkamp, Menno P. J. de Winther, Johan Kuiper, Ilze Bot, Bram Slütter

**Affiliations:** 1 Leiden Academic Centre for Drug Research, Division of Biotherapeutics, Leiden University, Leiden, the Netherlands; 2 Amsterdam University Medical Centers, University of Amsterdam, Experimental Vascular Biology, Department of Medical Biochemistry, Amsterdam Cardiovascular Sciences, Amsterdam Infection and Immunity, Amsterdam, the Netherlands; 3 Central Diagnostic Laboratory, University Medical Center, Utrecht University, Utrecht, the Netherlands; 4 Department of Surgery, Haaglanden Medisch Centrum Westeinde, The Hague, the Netherlands; 5 Department of Vascular Surgery, University Medical Centre Utrecht, Utrecht, the Netherlands

**Keywords:** Autoimmunity, Atherosclerosis

## Abstract

Atherosclerosis is a lipid-driven chronic inflammatory disease; however, whether it can be classified as an autoimmune disease remains unclear. In this study, we applied single-cell T cell receptor seqencing (scTCR-seq) on human carotid artery plaques and matched peripheral blood mononuclear cell samples to assess the extent of TCR clonality and antigen-specific activation within the various T cell subsets. We observed the highest degree of plaque-specific clonal expansion in effector CD4^+^ T cells, and these clonally expanded T cells expressed genes such as *CD69*, *FOS* and *FOSB*, indicative of recent TCR engagement, suggesting antigen-specific stimulation. CellChat analysis suggested multiple potential interactions of these effector CD4^+^ T cells with foam cells. Finally, we integrated a published scTCR-seq dataset of the autoimmune disease psoriatic arthritis, and we report various commonalities between the two diseases. In conclusion, our data suggest that atherosclerosis has an autoimmune compondent driven by autoreactive CD4^+^ T cells.

## Abstract (web summary)

Depuydt and Schaftenaar et al. profile the T cell clonality in patients with atherosclerosis by performing single-cell T cell receptor sequencing on carotid artery plaques and matched peripheral blood mononuclear cell samples. The analyses showed plaque-specific clonal expansion in effector CD4^+^ T cells, expressing genes indicative of exposure to activating antigens, thus suggesting that atherosclerosis has an autoimmune component driven by autoreactive CD4^+^ T cells.

## Main

Atherosclerosis is the major underlying pathology of acute cardiovascular events, such as myocardial infarction and stroke. It is characterized by accumulation of lipids and subsequent inflammation of the medium and large arteries. As low-density lipoprotein (LDL) particles are important instigators of atherosclerosis, cardiovascular disease (CVD) has primarily been treated as a lipid-driven disorder, with a treatment focus on lowering LDL cholesterol levels. Nonetheless, inflammation plays a critical role in perpetuating the growth and instability of atherosclerotic lesions, highlighted by the success of recent clinical trials with anti-inflammatory agents^1,2^. Elucidating the dominant inflammatory pathways that drive atherosclerosis may, therefore, allow identification of new druggable targets independent of cholesterol lowering.

Single-cell RNA sequencing (scRNA-seq) and mass cytometry have allowed detailed mapping of the leukocyte contents of atherosclerotic plaques^3,4^. These studies show that T cells are the largest leukocyte population and that the number of effector T cells within the lesion associates with plaque instability. In combination with previous murine work, this suggests that inflammatory processes inside the plaque are driven by T cells, and atherosclerosis could be considered an autoimmune-like disease. In support of that, autoreactive (LDL-specific) CD4^+^ T cells have previously been reported in human atherosclerotic lesions and have been identified in elevated levels in the circulation of patients with CVD^5–7^. Moreover, vaccination approaches aimed at the reduction of self-reactive T cells or induction of regulatory T (T_reg_) cells have shown promise in murine models of atherosclerosis^8,9^. However, when self-reactive CD4^+^ T cells are indeed the culprit T cells that propagate disease, clonal expansion and accumulation of these cells in the lesions is to be expected. Interestingly, recent work examining the T cell receptor (TCR) distribution in human coronary plaques showed primarily clonal expansion of CD8^+^ T cells inside the plaque and identified some of these TCRs to be specific for common viral antigens, such as influenza, cytomegalovirus (CMV) and severe acute respiratory syndrome coronavirus 2 (SARS-CoV-2)^10^. However, this work did not include patient-matched peripheral blood mononuclear cell (PBMC) controls, rendering it impossible to assess whether the virus-specific CD8^+^ T cells were specifically enriched in the plaque and/or had recently undergone antigen-specific interactions.

Here we present an approach to identify the T cell subsets that are specifically enriched in atherosclerotic lesions and whether these subsets underwent antigen-specific interaction in the plaque. We combine scRNA-seq and single-cell TCR sequencing (scTCR-seq) of human carotid plaques and matched PBMC samples. With this approach, we observed the highest degree of plaque-specific clonal expansion in both effector CD4^+^ T cells and, to a smaller extent, in the T_reg_ population. By integrating the data from our patients with atherosclerosis with the scTCR-seq data from patients with psoriatic arthritis (PSA), we show that atherosclerosis has major similarities with another prominent autoimmune disease. Thus, our data suggest that atherosclerosis is characterized by an autoimmune component driven by autoreactive CD4^+^ T cells.

## Results

### Signature of antigen-specific T cells in atherosclerosis

**Fig. 1:**
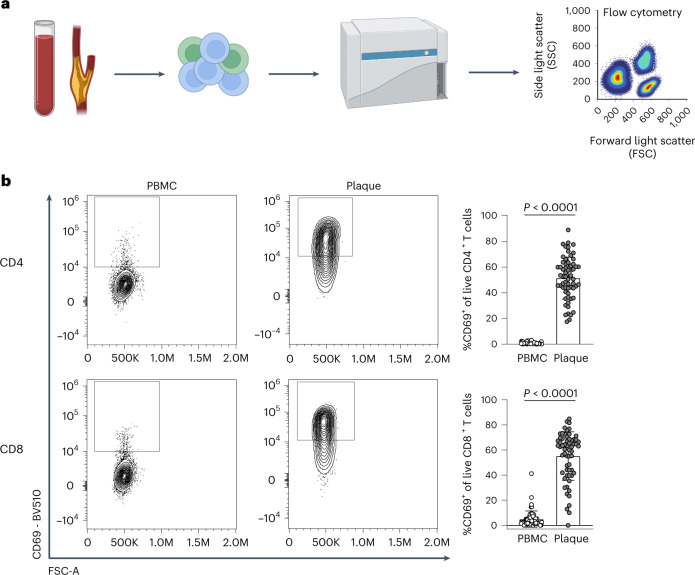
Significant increase in CD69^+^ T cells in the atherosclerotic plaque suggests an antigen-specific T cell response. **a**, Experimental setup: single cells from PBMC and plaque samples were stained with fluorescently labelled antibodies and measured through flow cytometry. **b**, Flow cytometry analysis of CD69 expression on PBMC and plaque live CD4^+^ and CD8^+^ T cells. *P* values are depicted in the figure panels. Data are presented as mean values ± s.d. PBMC *n* = 58; plaque *n* = 61. Statistical analyses were performed using an unpaired Mann–Whitney *t*-test. Source data

**Extended Data Fig. 1:**
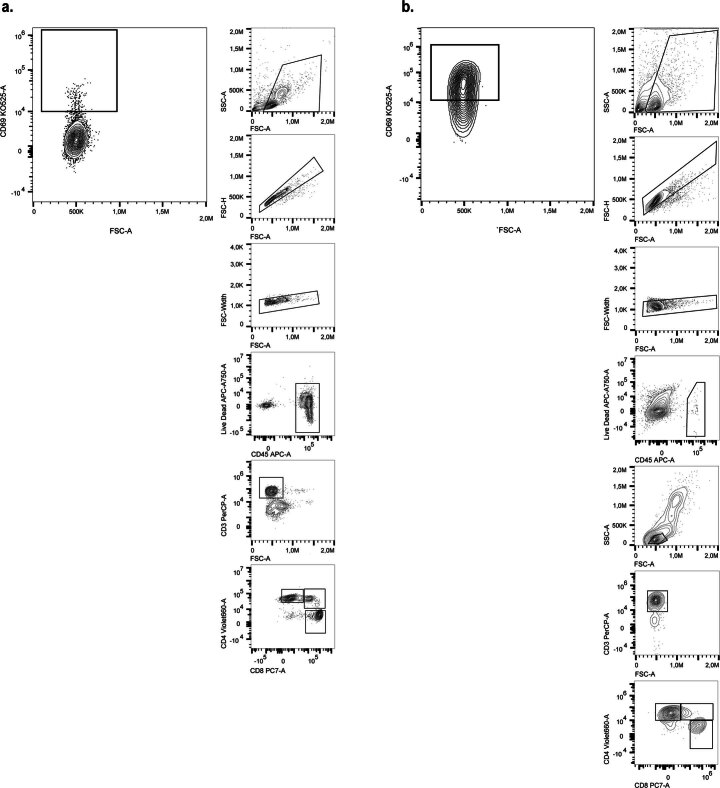
Gating strategy of flow cytometry of CD69^+^ T cells. **a**. Example of gating and gating ancestry of CD69^+^ T cells in PBMC. **b**. Example of gating and gating ancestry of CD69^+^ in the plaque.

Recent scRNA-seq studies in human atherosclerosis have shown a prominent accumulation of T cells in the plaque^3,4^. However, it remains unclear whether these T cells are bystanders or whether they actively contribute to lesion progression through antigen-specific activation. To examine potential recent antigen encounter and activation, CD69 expression was measured on the surface of both PBMCs and plaque T cells through flow cytometry (cohort 1; Fig. 1a and Supplementary Table 1). A significant increase in CD69^+^CD4^+^ (PBMC: 0.82% ± 0.71, plaque: 51.45% ± 16.39; *P* < 0.0001) and CD8^+^ T cells (PBMC: 4.95% ± 6.49, plaque: 55.20% ± 19.40; *P* < 0.0001) was observed in the plaque compared to PBMC (Fig. 1b and Extended Data Fig. 1a,b). Because CD69 is known to rapidly upregulate after TCR/HLA engagement on T cells^11^, these data suggest that T cells actively engage in TCR-specific interactions within the atherosclerotic plaque.

**Fig. 2:**
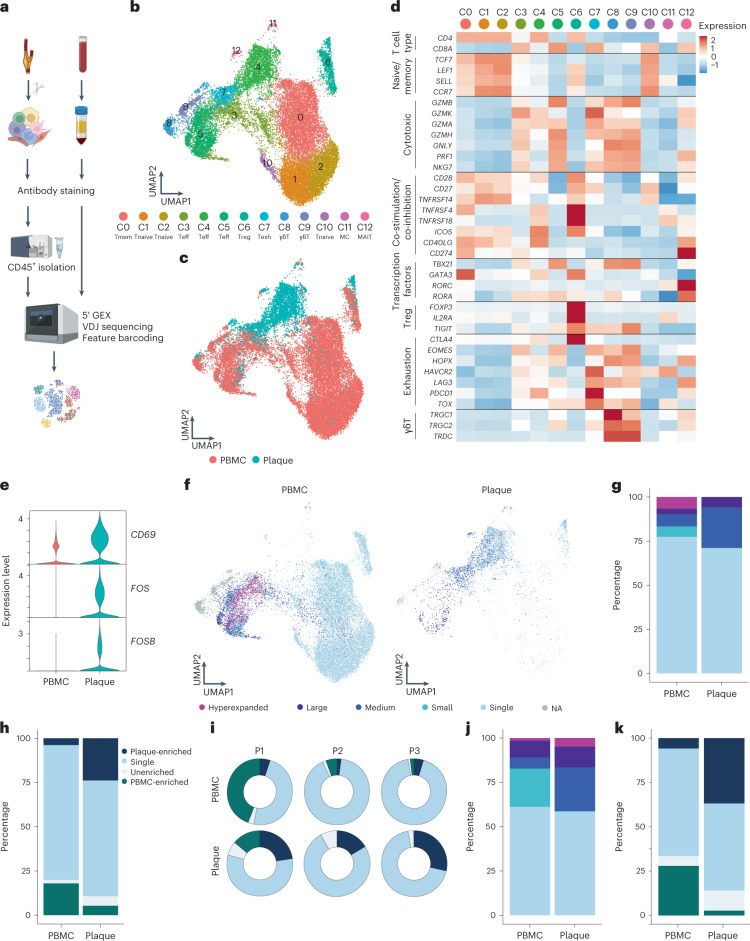
scTCR-seq reveals clonal expansion and antigen-specific activation of T cells in the plaque. **a**, Schematic overview of the study design. Human plaques were enzymatically digested, and live CD45^+^ cells were sorted using FACS. Matched blood samples were processed to isolate PBMCs. Both plaque cells and PBMCs were then further processed using 10x Genomics and sequenced. **b**, UMAP depicting 13 distinct T cell clusters resulting from unsupervised clustering (*n* = 24,443). **c**, UMAP showing contribution of PBMC or plaque to the T cell clusters. **d**, Heat map with average expression of T cell function-associated genes. **e**, Violin plot with expression of *CD69*, *FOS* and *FOSB* in PBMCs and plaque T cells. **f**, UMAP visualization of clonotype expansion levels among T cells between PBMC and plaque. **g**, Bar plot with quantification of clonal expansion levels between plaque and PBMC T cells. **h**, Bar plot with quantification of tissue enrichment scores of clonotypes. **i**, Circle plots depicting tissue enrichment scores of all T cells per tissue and per patient. **j**, Bar plot with quantification of clonal expansion levels between PBMC and plaque T cells of bulk TCR-seq data (cohort 3, *n* = 10). **k**, Bar plot with quantification of tissue enrichment scores of bulk TCR-seq data (cohort 3). Clonotype expansion levels: Single (one occurrence), Small (≤0.1%), Medium (>0.1% and ≤1%), Large (>1% and ≤10%) and Hyperexpanded (>10%), percentage of all T cells. Tissue enrichment scores: Plaque-enriched (frequency expanded clone higher in plaque versus PBMC), Single (one occurrence), Unenriched (frequency expanded clone similar in PBMC versus plaque) and PBMC-enriched (frequency expanded clone higher in PBMC versus plaque). Source data

**Extended Data Fig. 2:**
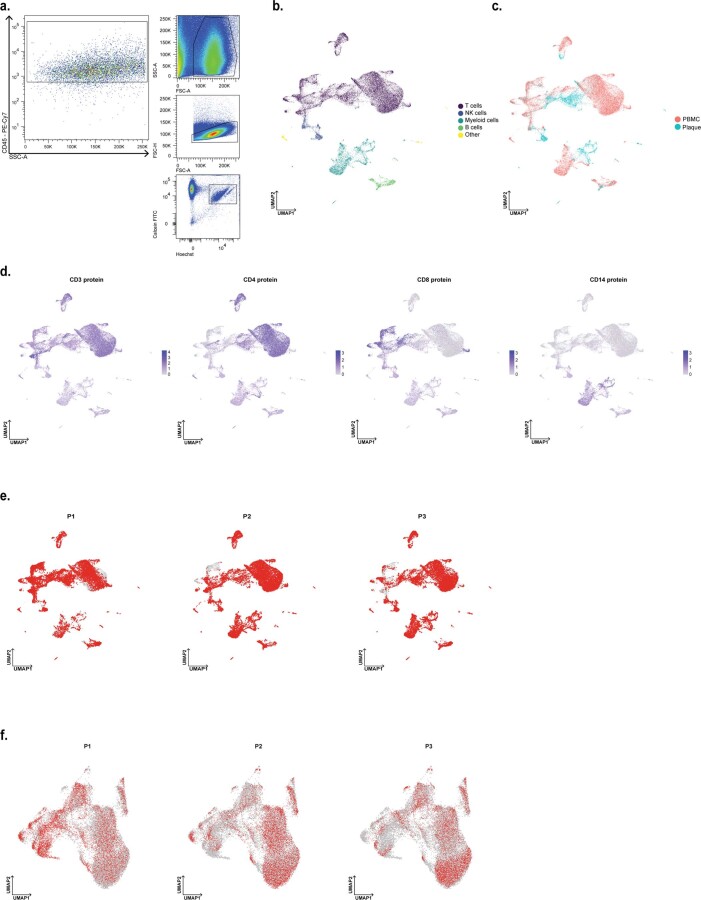
Single-cell RNA sequencing of PBMC and live CD45^+^ plaque cells. **a**. Gating strategy used for fluorescent-activated cell sorting (FACS) to isolate plaque live CD45^+^ cells for 10X Genomics and sequencing. **b**. UMAP projection of all PBMC and plaque cells, depicting multiple leukocyte types (n = 33249). **c**. UMAP visualization of tissue distribution of PBMC and plaque cells. **d**. UMAP projection of protein expression of CD3, CD4, CD8 and CD14 on all PBMC and plaque cells. **e**. Patient contribution to UMAP of all PBMC and plaque cells. Red dots indicate cells that are retrieved from the abovementioned patient. **f**. Patient contribution to UMAP of PBMC and plaque T cells. Red dots indicate cells that are retrieved from the abovementioned patient.

However, CD69 expression may also indicate the presence of resident memory T cells or may be upregulated by exposure to type I interferon (IFN)^12,13^. To determine whether the elevated CD69 expression was due to antigen-specific interactions in the plaque, we aimed to assess whether these T cells were clonally expanded as well. We combined scRNA-seq with scTCR-seq on paired PBMCs and carotid artery plaques from three male patients (cohort 2; Supplementary Table 1). The plaques were enzymatically digested, and live CD45^+^ cells were isolated by fluorescence-activated cell sorting (FACS) (Extended Data Fig. 2a). Both PBMCs and plaque cells were stained for CD3, CD4, CD8 and CD14 on a protein level with feature barcoding to properly distinguish between myeloid and T cell subsets on both RNA and protein level. All cells were subsequently processed with droplet-based single-cell 5′ RNA sequencing (10x Genomics) and sequenced (Fig. 2a). Unsupervised clustering revealed clusters consisted of T cells, natural killer (NK) cells, myeloid cells and B cells, originating from both PBMCs and plaque cells and with limited interpatient variability (Extended Data Fig. 2b–e). We did not further characterize all non-T cells, as we specifically focused on characterizing T cells to assess their clonal expansion in atherosclerosis. Therefore, all T cells were selected based on both RNA and protein expression, and, subsequently, unsupervised clustering was performed independent of the variable TCR genes to prevent clustering based on clonality (Methods). Subclustering of both PBMCs and plaque T cells revealed 13 distinct T cell subsets (Fig. 2b,c and Extended Data Fig. 2f). Within the T cells, we observed one memory (C0) and three naive (C1, C2 and C10) T cell clusters based on different expression levels of *TCF7*, *LEF1*, *SELL* and *CCR7* (Fig. 2b,d and Supplementary Table 2). Furthermore, three effector T cell clusters (C3, C4 and C5) were detected, expressing a multitude of different cytotoxic genes, such as *GZMB*, *GZMK* and *GZMA* (Fig. 2b,d and Supplementary Table 2). A T_reg_ cluster was defined based on expression of *FOXP3*, *IL2RA* and *TIGIT* (C6; Fig. 2b,d and Supplementary Table 2)^14^. In addition, an exhausted T cell cluster characterized by expression of *HAVCR2*, *PDCD1* and *TOX*^15,16^ (C7; Fig. 2b,d and Supplementary Table 2) and two γδ-T cell clusters expressing *TRGC1*, *TRGC2* and *TRDC* (C8 and C9; Fig. 2b,d and Supplementary Table 2) were detected. Lastly, we observed two small clusters consisting of mast cells (C11; Fig. 2b and Supplementary Table 2) and mucosal-associated invariant T (MAIT) cells (C12; Fig. 2b,d and Supplementary Table 2).

Next, we compared expression of *CD69* as well as *FOS* and *FOSB* genes, which are also upregulated downstream of TCR signalling^17^, between plaque and blood. In line with the increased CD69^+^ protein expression measured through flow cytometry, all three genes showed an increased mRNA expression in plaque T cells compared to their PBMC counterparts (Fig. 2e). Subsequently, we applied VDJ sequencing to map paired α-chains and β-chains of the TCR and to define the clonal composition of the paired PBMCs and plaque T cells. Clonal expansion levels were calculated to indicate the clonotype abundance as percentage of the total measured TCRs per patient, per tissue (Fig. 2f and Methods). ‘Single’ represents a single clonotype occurrence. Expanded T cells were divided into multiple categories characterized by increasing frequencies of clonotype occurrences, labelled as ‘Small’, ‘Medium’, ‘Large’ and ‘Hyperexpanded’.

**Extended Data Fig. 3:**
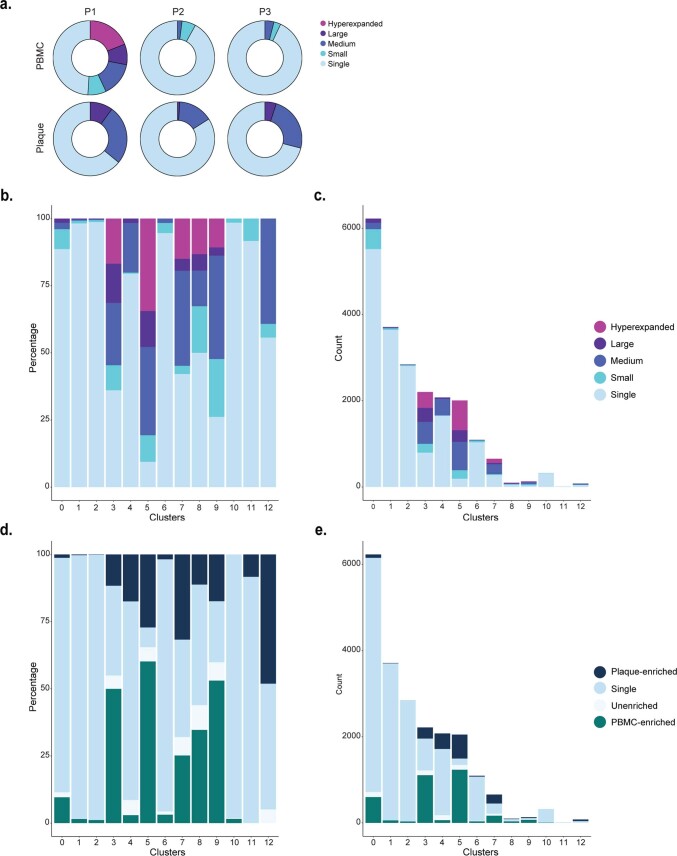
Distribution of clonal expansion levels and tissue-enrichment scores in T cell clusters. **a**. Circle plots depicting clonal expansion levels of all T cells per tissue and per patient. **b**. Barplot with relative quantification of clonal expansion levels per cluster. **c**. Barplot with absolute quantification of clonal expansion levels per cluster. **d**. Relative quantification of tissue enrichment scores per cluster. **e**. Barplot with absolute quantification of tissue enrichment scores per cluster. Clonotype expansion levels: Single (one occurrence), Small (≤0.1%), Medium (>0.1 & ≤1%), Large (>1 & ≤10%), Hyperexpanded (>10%), percentage of all T cells. Tissue enrichment scores: Plaque-enriched (Frequency expanded clone higher in Plaque vs. PBMC), Single (one occurrence), Unenriched (Frequency expanded clone similar in PBMC vs. Plaque), PBMC-enriched (Frequency expanded clone higher in PBMC vs Plaque). Source data

**Extended Data Fig. 4:**
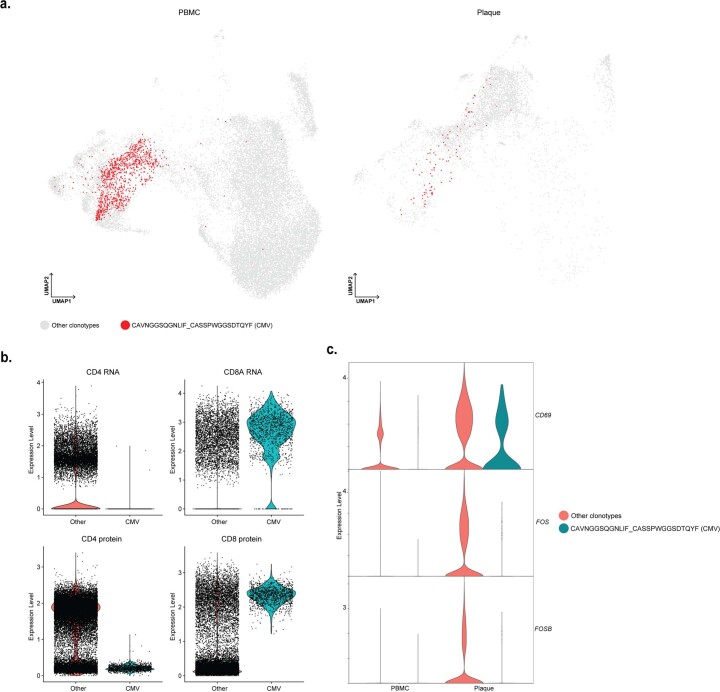
Hyperexpanded CMV clonotype does not show signs of recent T cell activation. **a**. UMAP projection of clonotype CAVNGGSQGNLIF_CASSPWGGSDTQYF (CMV) on PBMC and plaque T cells. Red dots indicate T cells with clonotype CAVNGGSQGNLIF_CASSPWGGSDTQYF, grey dots indicate T cells with other clonotypes. **b**. Violin plots projecting gene expression of *CD4*, *CD8A* and protein expression of CD4 and CD8 split by T cells with and without clonotype CAVNGGSQGNLIF_CASSPWGGSDTQYF. **c**. Violin plots projecting expression of *CD69*, *FOS* and *FOSB* split by tissue and presence of clonotype CAVNGGSQGNLIF_CASSPWGGSDTQYF.

**Extended Data Fig. 5:**
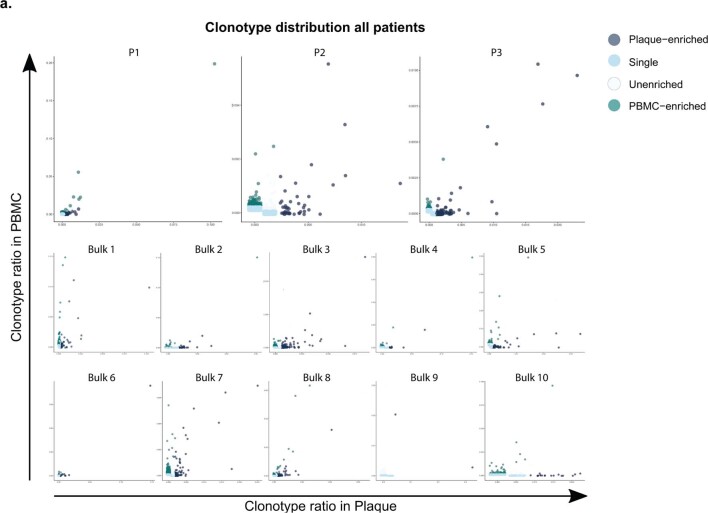
Distribution of expanded TCRs in scTCRseq and TCRβ bulk data sets. **a**. Scatterplot projecting frequencies of clonotypes and their tissue enrichment scores in PBMC and plaque per patient of the single-cell TCR sequencing dataset (Cohort 2) and the TCRβ bulk sequencing data set (Cohort 3). Tissue enrichment scores: Plaque-enriched (Frequency expanded clone higher in Plaque vs. PBMC), Single (1 occurrence), Unenriched (Frequency expanded clone similar in PBMC vs. Plaque), PBMC-enriched (Frequency expanded clone higher in PBMC vs Plaque).

Taken together, a small increase in the percentage of total expanded T cells is observed in the plaque compared to PBMCs (PBMC 23% versus plaque 29%; Fig. 2f,g, Extended Data Fig. 3a–c and Supplementary Table 3). One clonotype, originating from patient 1, was defined as Hyperexpanded in the PBMC and Large in the plaque. The TCRα sequence of this clonotype matched with a TCRα sequence previously associated with CMV in the VDJdb database (https://vdjdb.cdr3.net/)^18^. The CD8^+^ T cell-specific clonotype, however, was only expressed in T cells that had little expression of *CD69*, *FOS* and *FOSB*, suggesting that this was not an active viral infection (Extended Data Fig. 4a–c). In addition, the tissue enrichment of clonotypes was assessed to investigate whether certain clonotypes specifically accumulated within either of the tissues or whether the clonotype abundance was unaffected by the location. T cells with clonotypes more present in the PBMC were identified as PBMC-enriched and vice versa for plaque-enriched T cells. Indeed, within the plaque, an increased percentage of plaque-enriched T cells was observed in all patients, suggesting a potential plaque-restricted antigen-induced clonal expansion (Fig. 2h,i, Extended Data Fig. 3d,e and Supplementary Table 3). To confirm these findings, bulk TCRβ sequencing was performed on matched blood and plaque T cells from ten patients (cohort 3; Supplementary Table 1). Both clonal expansion levels and tissue enrichment were similar between TCRβ bulk sequencing and the scTCR-seq data (Fig. 2j,k and Extended Data Fig. 5a).

### Increased percentage of expanded CD8^+^ T cells in PBMCs

**Fig. 3:**
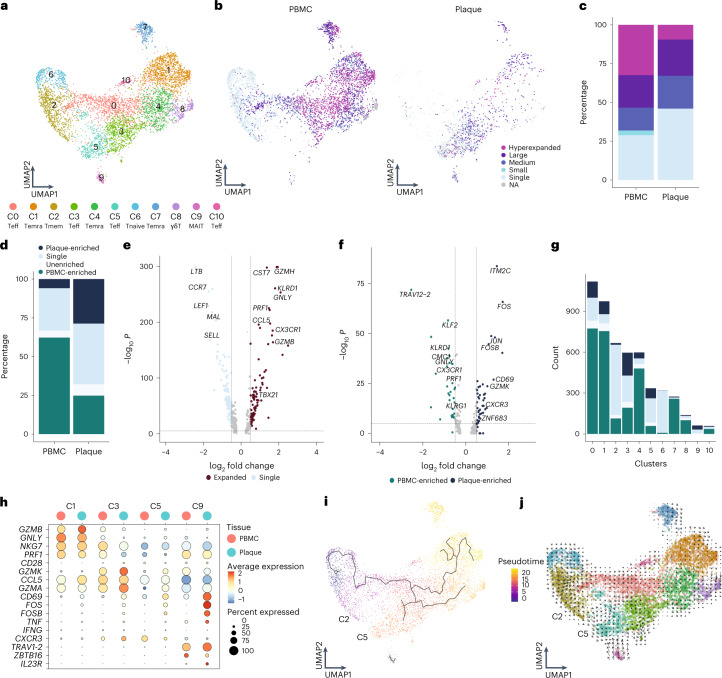
Limited clonal expansion in plaque CD8^+^ T cells compared to PBMCs. **a**, UMAP visualization of unsupervised clustering revealed 11 distinct CD8^+^ T cell populations (*n* = 5,730). **b**, UMAP visualization of different levels of clonotype expansion among CD8^+^ T cells between PBMC and plaque. **c**, Quantification of clonal expansion levels between PBMC and plaque CD8^+^ T cells. **d**, Quantification of tissue enrichment scores of clonotypes in CD8^+^ T cells of PBMC and plaque. **e**, Volcano plot with differentially expressed genes between CD8^+^ T cells with single clonotypes and all expanded clonotypes (Small–Large). Genes were considered significant with *P* < 1 × 10^−6^ and a fold change of 0.5. For all volcano plots, Bonferroni-corrected *P* values were calculated based on the total number of genes in the dataset. **f**, Volcano plot with differentially expressed genes of PBMC-enriched versus plaque-enriched CD8^+^ T cells. Genes were considered significant with *P* < 1 × 10^−6^ and a fold change of 0.5. **g**, Bar plot with quantification of tissue enrichment score of individual CD8^+^ T cell clusters. **h**, Dot plot of average expression of upregulated genes in clusters 1, 3, 5 and 9. **i**, UMAP visualization of pseudotime analysis of CD8^+^ T cells. C2 indicates cluster 2; C5 indicates cluster 5. **j**, UMAP visualization of RNA velocity analysis of CD8^+^ T cells. Clonotype expansion levels: Single (one occurrence), Small (≤0.1%), Medium (>0.1% and ≤1%), Large (>1% and ≤10%) and Hyperexpanded (>10%), percentage of all CD8^+^ T cells. Tissue enrichment scores: Plaque-enriched (frequency expanded clone higher in plaque versus PBMC), Single (one occurrence), Unenriched (frequency expanded clone similar in PBMC versus plaque) and PBMC-enriched (frequency expanded clone higher in PBMC versus plaque).

**Extended Data Fig. 6:**
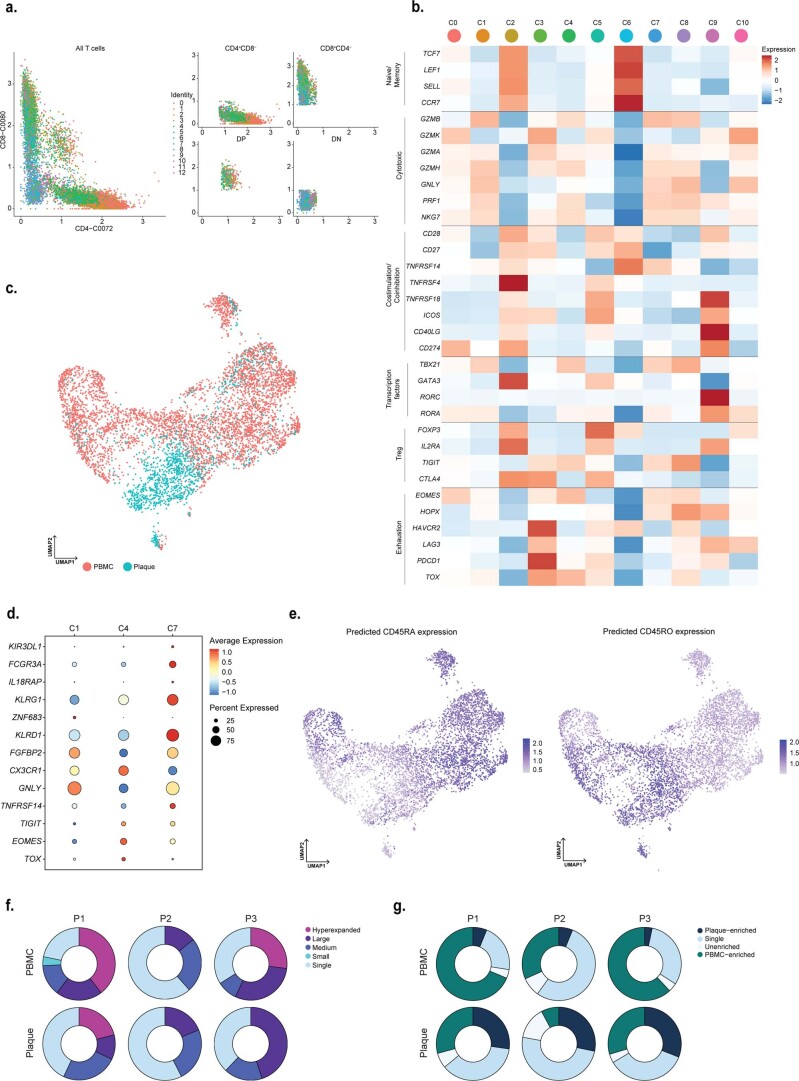
CD8^+^ T cell marker genes and tissue distribution. **a**. CD4 and CD8 protein expression on all T cells colored by cluster ID. Visualization of selection of CD4^+^CD8^−^, CD4^−^CD8^+^, double positive (DP) and double negative (DN) cells. CD4^+^CD8^−^ cells were used for subclustering of CD4^+^ T cells. CD4^−^CD8^+^ cells were used for subclustering of CD8^+^ T cells. **b**. UMAP projection of tissue distribution of PBMC and plaque CD8^+^ T cells. **c**. Heatmap with expression of T cell function-associated genes in CD8^+^ T cell clusters. **d**. Dot plot visualization of a selection of differentially regulated genes, excluding TCR complex genes, between clusters 1, 4 and 7. **e**. Predicted expression of CD45RA and CD45RO based on mapping the data with Seurat multimodal reference mapping. **f**. Circle plots depicting clonal expansion levels of CD8^+^ T cells per tissue and per patient. **g**. Circle plots depicting tissue-enrichment scores of CD8^+^ T cells per tissue and per patient. Clonotype expansion levels: Single (one occurrence), Small (≤0.1%), Medium (>0.1 & ≤1%), Large (>1 & ≤10%), Hyperexpanded (>10%), percentage of all CD8^+^ T cells. Tissue enrichment scores: Plaque-enriched (Frequency expanded clone higher in Plaque vs. PBMC), Single (one occurrence), Unenriched (Frequency expanded clone similar in PBMC vs. Plaque), PBMC-enriched (Frequency expanded clone higher in PBMC vs Plaque). Source data

To properly isolate CD4^+^ and CD8^+^ T cells for further analysis, a selection was made of CD4^+^ and CD8^+^ single-positive T cells based on expression of these proteins as measured by feature barcoding (Extended Data Fig. 6a). Subclustering of CD8^+^ T cells resulted in 11 distinct subsets. Most CD8^+^ T cells had an activated phenotype as indicated by expression of multiple genes with a cytotoxic signature. One naive (C6) and one memory (C2) cluster were mainly detected in the PBMC (*TCF7*, *LEF1*, *SELL* and *CCR7*; Fig. 3a, Extended Data Fig. 6b,c and Supplementary Table 2). Four effector clusters were characterized, of which C0 and C10 mostly reside in the PBMC and C3 and C5 predominantly in the plaque. C0, C3 and C10 expressed a multitude of different cytotoxic genes, including *GZMK* and *GZMA*, at different levels. C5 was characterized by expression of *CD69*, *FOS* and *FOSB* (Fig. 3a, Extended Data Fig. 6b,c and Supplementary Table 2). Furthermore, three terminally differentiated effector memory T cell (T_EMRA_) clusters were defined by expression of, for example, *GZMB*, *PRF1* and *NKG7* and lack of *CD27* and *CD28* (C1, C4 and C7; Fig. 3a, Extended Data Fig. 6b,c and Supplementary Table 2). T_EMRA_ clusters were primarily associated with a gradual increase in expression of, among others, *KLRD1*, *KLRG1* and *FCGR3A*, indicating various stages of terminal differentiation (Extended Data Fig. 6d). Using Seurat multimodal reference mapping, which maps your dataset to a large PBMC dataset with feature barcoding data, expression of CD45RA and CD45RO could be predicted. Indeed, T_EMRA_ subsets were predicted to express CD45RA, whereas the effector T cells were predicted to be CD45RO^+^ (Extended Data Fig. 6e). Finally, a cluster of γδ-T cells (C8) and a cluster of MAIT cells (C9) were detected within the CD8^+^ T cell subsets (Fig. 3a, Extended Data Fig. 6a,b and Supplementary Table 2). Subsequently, clonal expansion levels were examined and quantified within the CD8^+^ T cells in PBMC and plaque. A large percentage of clonally expanded CD8^+^ T cells was detected in the plaque; however, a higher percentage of expanded CD8^+^ T cells was detected in the PBMC (Fig. 3b,c, Extended Data Fig. 6f and Supplementary Table 3). Nevertheless, within the plaque, most expanded CD8^+^ T cells remained plaque enriched (Fig. 3d, Extended Data Fig. 6g and Supplementary Table 3). Expanded CD8^+^ T cells showed upregulation of multiple genes involved in CD8 cytotoxicity—for example, *GZMH*, *KLRD1*, *PRF1* and *GZMB* (Fig. 3e). Interestingly, when comparing PBMC-enriched versus plaque-enriched CD8^+^ T cells, PBMC-enriched cells expressed cytotoxic genes, such as *GNLY*, *PRF1* and members of the killer cell lectin-like subfamily (*KLRG1* and *KLRD1*), whereas plaque-enriched CD8^+^ T cells seemed to have experienced recent antigen-induced TCR activation (Fig. 3f). To further illustrate the plaque-expanded CD8^+^ T cell clusters, we selected C1, C3, C5 and C9, which had relatively the most plaque-enriched expansion (Fig. 3g). C1, C3 and C5 all expressed a multitude of cytotoxic genes. C1 highly expressed *NKG7*, *GNLY* and *GZMB*, of which the latter was increased in plaque, whereas C3 and C5 had increased expression of *GZMA* and *GZMK* in the plaque. C5 plaque T cells had the highest expression of *CD69*, *FOS* and *FOSB*. Finally, MAIT cells (C9) showed high expression of genes unique for this cell type (*TRAV1-2*, *ZBTB16* and *IL23R*)^19^ and of TCR activation genes. To identify potential dynamics of different CD8^+^ populations, we applied lineage tracing analyses using Monocle3 and RNA velocity. RNA velocity shows that, within the CD8^+^ clusters, cells tend to be less prone to switch into another subset. A small trajectory appeared between the memory CD8^+^ T cells (C2) and the antigen-experienced effector T cells (C5), yet this was not clearly retrieved with pseudotime analysis (Fig. 3i,j).

### Increased percentage of expanded CD4^+^ T cells in plaque

**Fig. 4:**
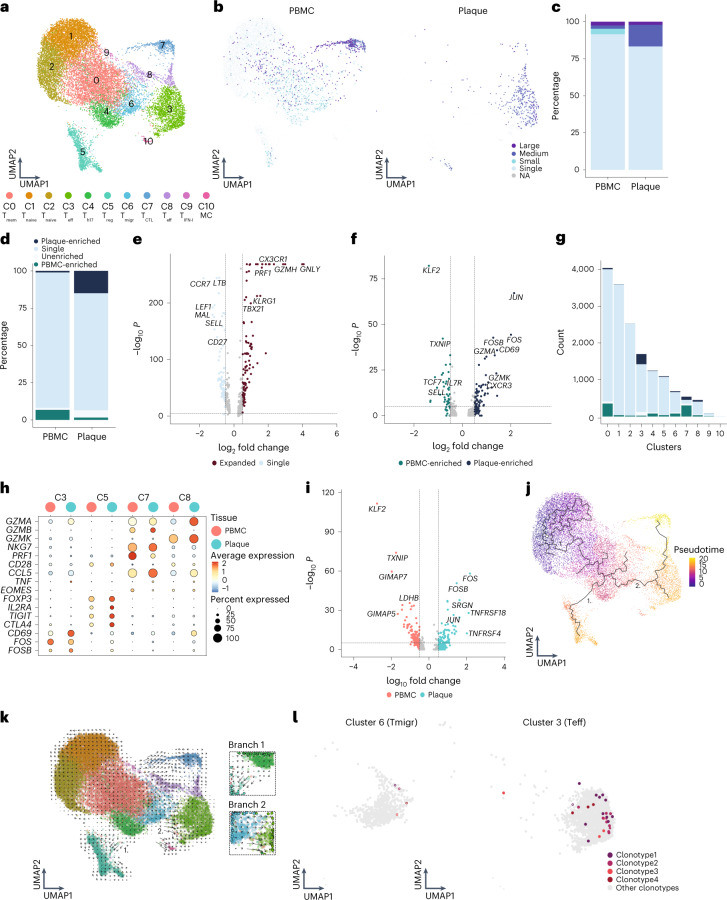
Increased percentage of expanded and plaque-enriched CD4^+^ T cells in the atherosclerotic plaque. **a**, UMAP visualization of unsupervised clustering revealed 11 distinct CD4^+^ T cell populations (*n* = 17,073). **b**, UMAP visualization of different levels of clonotype expansion among CD4^+^ T cells between PBMC and plaque. **c**, Bar plot with quantification of clonal expansion levels between PBMC and plaque CD4^+^ T cells. **d**, Bar plot with quantification of tissue enrichment scores of clonotypes in CD4^+^ T cells of PBMC and plaque. **e**, Volcano plot with differentially expressed genes between CD4^+^ T cells with single clonotypes and all expanded clonotypes (Small–Large). Genes were considered significant with *P* <1 × 10^−6^ and a fold change of 0.5. For all volcano plots, Bonferroni-corrected *P* values were calculated based on the total number of genes in the dataset. **f**, Volcano plot with differentially expressed genes of PBMC-enriched versus plaque-enriched CD4^+^ T cells. Genes were considered significant with *P* <1 × 10^−6^ and a fold change of 0.5. **g**, Bar plot with quantification of tissue enrichment score of individual CD4^+^ T cell clusters. **h**, Dot plot of average expression of upregulated genes in clusters 3, 5, 7 and 8. **i**, Volcano plot with differentially expressed genes between T_reg_ cells in PBMC and plaque. Genes were considered significant with *P* <1 × 10^−6^ and a fold change of 0.5. **j**, UMAP visualization of pseudotime analysis of CD4^+^ T cells. Two branches of the analysis are indicated with 1 and 2. **k**, UMAP visualization of RNA velocity analysis of CD4^+^ T cells with close-up of branches 1 and 2. **l**, UMAP visualization of four overlapping clonotypes between cluster 6 and cluster 3. Open circles indicate PBMC CD4^+^ T cells; closed circles indicate plaque CD4^+^ T cells. Clonotype expansion levels: Single (one occurrence), Small (≤0.1%), Medium (>0.1% and ≤1%), Large (>1% and ≤10%), percentage of all CD4^+^ T cells. Tissue enrichment scores: Plaque-enriched (frequency expanded clone higher in plaque versus PBMC), Single (one occurrence), Unenriched (frequency expanded clone similar in PBMC versus plaque), PBMC-enriched (frequency expanded clone higher in PBMC versus plaque).

**Extended Data Fig. 7:**
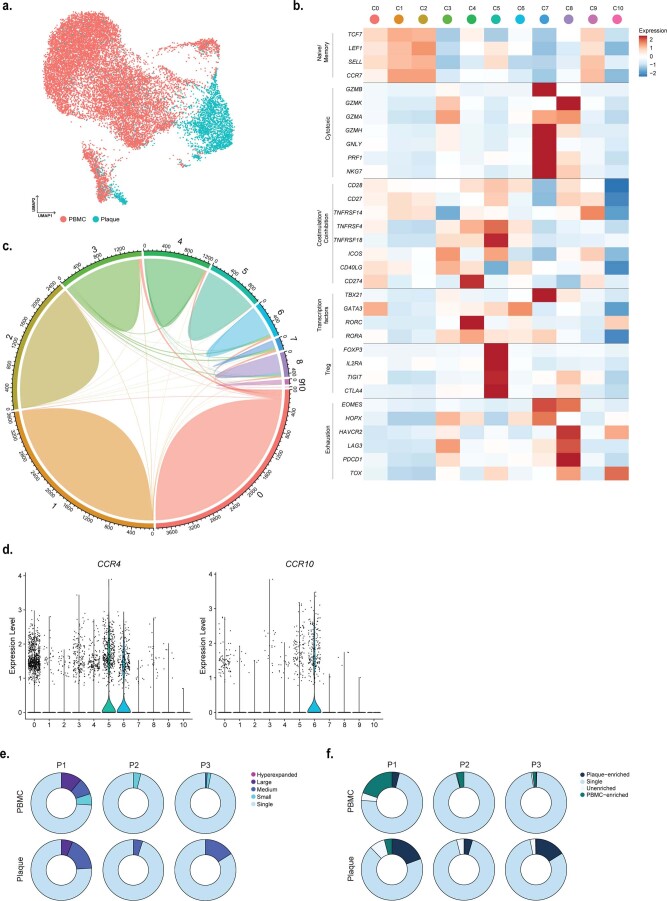
CD4^+^ T cell marker genes and tissue distribution. **a**. UMAP visualization of tissue distribution of PBMC and plaque CD4^+^ T cells. **b**. Heatmap with expression of T cell function-associated genes in CD4^+^ T cell clusters. **c**. Circle plot visualizing the overlap of clonotypes between all CD4^+^ clusters. Each color represents a different cluster. Axis indicates the number of TCRs. Line thickness indicates the number of overlapping clonotypes. **d**. Violin plots depicting expression of CCR4 and CCR10 in CD4+ T cell clusters. **e**. Circle plots depicting clonal expansion levels of CD4^+^ T cells per tissue and per patient. **f**. Circle plots depicting tissue-enrichment scores of CD4+ T cells per tissue and per patient. Clonotype expansion levels: Single (one occurrence), Small (≤0.1%), Medium (>0.1 & ≤1%), Large (>1 & ≤10%), Hyperexpanded (>10%), percentage of all CD4^+^ T cells. Tissue enrichment scores: Plaque-enriched (Frequency expanded clone higher in Plaque vs. PBMC), Single (one occurrence), Unenriched (Frequency expanded clone similar in PBMC vs. Plaque), PBMC-enriched (Frequency expanded clone higher in PBMC vs Plaque). Source data

Unsupervised clustering revealed 11 subsets of CD4^+^ T cells (Fig. 4a). As previously described, CD4^+^ T cell clusters are mainly defined by a shift in activation status^3,4^. Two naive T cell clusters (C1 and C2) and a memory T cell cluster (C0) were mainly detected within the PBMC (Fig. 4a, Extended Data Fig. 7a,b and Supplementary Table 2). Furthermore, a T-helper (T_h_) 17-like cluster (C4) expressing *RORC*, *RORA* and *CCR6*, as well as a T_reg_ cluster (C5; Fig. 4a, Extended Data Fig. 7b and Supplementary Table 2), were identified. Whereas T_reg_ cells were found in both PBMC and plaque, T_h17_-like cells were mainly detected in PBMC (Extended Data Fig. 7c). A T cell cluster with genes involved in cell migration (T_migr_, C6) mainly resided in PBMC (Supplementary Table 2). Two different effector subsets were characterized, of which one was more plaque specific with high expression of *CD69*, *FOS*, *JUN* and *GZMA* (C3), and one was found in both tissues specifically enriched for *GZMK* (C8; Fig. 4a, Extended Data Fig. 7a,b and Supplementary Table 2). Moreover, a cytotoxic CD4^+^ T cell cluster, which resembled the previously described CD4^+^CD28^null^ cells^3,20,21^, was defined by expression of *GZMB* and *PRF1* and lack of *CD28* and was found in both PBMC and plaque (Fig. 4a, Extended Data Fig. 6a,b and Supplementary Table 2). Finally, a cluster of T cells was observed in the PBMCs that expressed genes involved in IFN I signalling and a small mast cell cluster in the plaque (Fig. 4a and Supplementary Table 2). Subsequently, CD4^+^ T cell clonality was assessed. Clonal expansion levels were projected on the CD4^+^ T cell uniform manifold approximation and projection (UMAP) and quantified. In line with a recent study by Chowdhury et al.^10^, the percentage of clonal expanded CD8^+^ T cells in the plaque is larger than those in CD4^+^ T cells. However, in contrast to CD8^+^ T cells, a marked increase in the percentage of expanded CD4^+^ T cells in the plaque was revealed compared to the PBMCs (Fig. 4b,c, Extended Data Fig. 7e and Supplementary Table 3). Furthermore, the expanded clonotypes in the plaque CD4^+^ T cells were mostly plaque enriched (Fig. 4d, Extended Data Fig. 7f and Supplementary Table 3). When comparing expanded CD4^+^ T cells to their single counterparts with a unique clonotype, upregulation of genes involved in T cell activation and cytotoxicity, such as *GNLY*, *GZMH*, *PRF1* and *CX3CR1*, were particularly observed in the expanded T cells, whereas single T cells expressed genes upregulated in naive and memory T cells (*CCR7*, *LTB*, *LEF1*, *SELL* and *CD27*) (Fig. 4e). Interestingly, when comparing clonally expanded PBMC-enriched versus the plaque-enriched expanded CD4^+^ T cells, plaque-enriched CD4^+^ T cells showed enhanced expression of genes upregulated shortly after antigen-specific TCR interaction (*JUN*, *CD69*, *FOS* and *FOSB*) (Fig. 4f), suggesting that there are CD4^+^ T cells that undergo antigen-specific interactions in the plaque. Next, we quantified the absolute number of plaque-enriched clones per CD4^+^ T cell cluster (Fig. 4g), which revealed cluster C3 as the major contributor in absolute number of plaque-specific clonally expanded T cells. Furthermore, C7 and C8 consisted of a relatively large number of plaque-enriched clones compared to the other CD4^+^ T cell clusters. The C7 cluster, characterized by an increase in cytotoxic genes, including *GZMB*, *NKG7* and *PRF1*, has little to no expression of *CD69*, *FOS* and *FOSB*, indicating that, although these cells have substantial expanded clonotypes, they do not express genes involved in antigen-induced activation (Fig. 4h). The effector populations C3 and C8 displayed increased expression of TCR proximal genes *CD69*, *FOS* and *FOSB*. Interestingly, whereas we did not observe increased accumulation of clonally expanded T_reg_ cells (C5) in plaque, we did observe upregulation of *FOS*, *FOSB* and *JUN* in plaque-derived T_reg_ cells compared to PBMC-derived T_reg_ cells, suggesting that T_reg_ cells are encountering antigen in the plaque. Expression of various functional T_reg_ markers (*FOXP3*, *IL2RA*, *TIGIT*, *CTLA4* and *TNFRSF4* (OX40) and *TNFRSF18* (GITR)) in the plaque compared to the PBMC indicated increased activity of T_reg_ cells (Fig. 4h,i).

To identify the origin of the antigen-specific effector CD4^+^ T cell subsets in the plaque, we applied lineage tracing analyses to define the dynamics of the different CD4^+^ T cell populations. Pseudotime analysis using Monocle3 showed a trajectory ranging from naive T cells toward either the T_reg_ cells (branch 1) or the effector T cell population (branch 2) (Fig. 4j). The first pseudotime branch directing toward T_reg_ cells is projected through the T_h17_-like CD4^+^ T cell cluster, potentially suggesting a plasticity between both subtypes. However, if the complementary RNA velocity analysis is assessed (time-resolved analysis based on spliced and unspliced mRNA^22^), the T_reg_ cluster does not seem to be derived from the T_h17_-like cells (branch 1; Fig. 4k). Moreover, T_reg_ cells in tissue also cluster further away from the circulating T_h17_-like cells compared to the PBMC T_reg_ cells, indicating that the plaque environment is less likely to induce a phenotype switch from T_reg_ to T_h17_. In addition, no overlapping clonotypes were found between both clusters, and *FOXP3* and *RORC* did not co-express (Extended Data Fig. 7b,c), suggesting that, in our dataset, we were not able to detect the previously described T_reg_/T_h17_ plasticity^23^. Looking at the other branch in both pseudotime analysis and RNA velocity (branch 2), a clear path ranging from the T_migr_ cluster (C6) toward the CD69^+^ T_eff_ cluster (C3) was observed. Their migratory phenotype, highlighted by expression of *CCR4* and *CCR10* previously described to be expressed on infiltrating T cells in the inflamed skin^24^, suggests that this T_migr_ subset could be the precursor population for the antigen-specific CD4^+^ T cells in the plaque (Extended Data Fig. 7d). Indeed, when comparing overlap in TCR sequence between the different CD4^+^ subpopulations, 37 clonotypes overlapped between both cluster C6 and cluster C3. Within the top five most expanded clonotypes, four plaque-enriched clonotypes were detected and exhibited marked expansion in C3 compared to C6, further confirming our hypothesis that the clonally expanded T_eff_ cells could originate from the circulating migratory T cell subset (Fig. 4l and Extended Data Fig. 7c).

### TREM2^+^ macrophages can activate antigen-induced CD4^+^ T cells

**Fig. 5:**
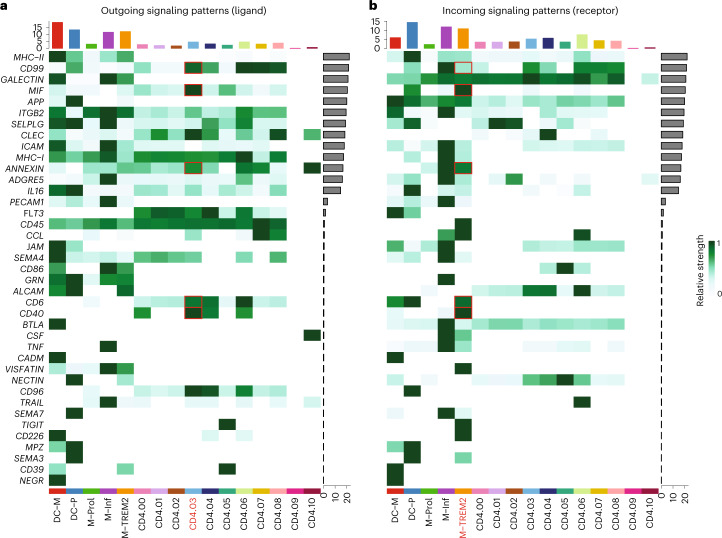
Enriched interaction pathways between CD4^+^ T_eff_ cells and TREM^hi^ macrophages. Heat maps displaying outgoing (ligand) (**a**) and incoming (receptor) (**b**) signalling patterns of pathways describing potential ligand–receptor interactions. Scale above the heat map indicates the relative signalling strength of a cell cluster based on all signalling pathways displayed in the heat map. Grey bars to the right of the heat map show the total signalling strength of a pathway in all cell clusters. The relative signalling strength is indicated by ranging colour from white (low) to green (high). All cells included in these graphs originate from the plaque.

**Extended Data Fig. 8:**
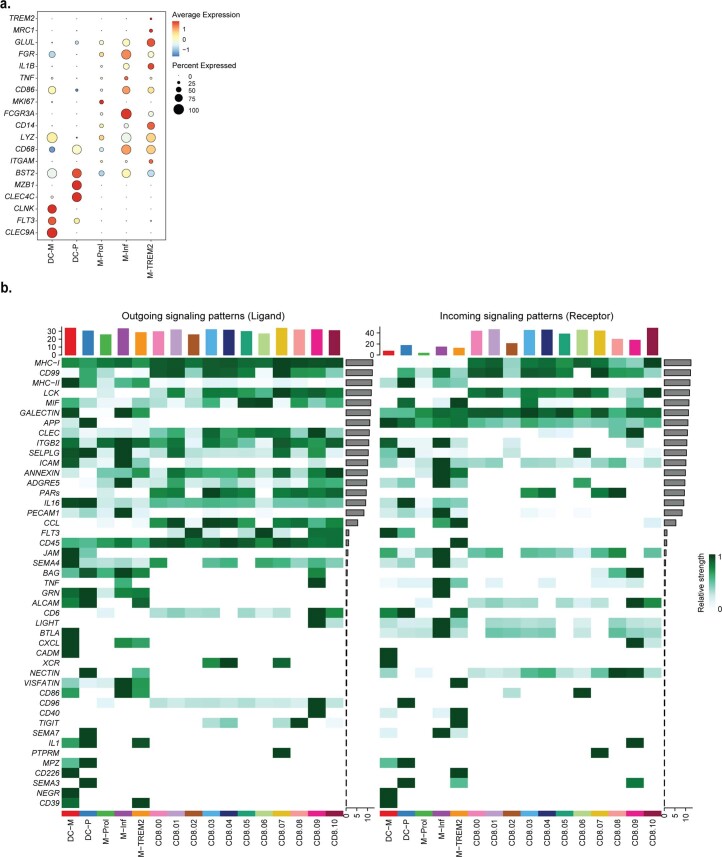
CellChat interaction pathways between CD8^+^ T cells and myeloid cells. **a**. Dotplot displaying average expression of genes describing the different dendritic cell and macrophage clusters. DC-M indicates myeloid-derived dendritic cell (DC); DC-P indicates plasmacytoid DC; M-PROL indicates proliferating macrophages; M-Inf indicates inflammatory macrophage; M-TREM2 indicates TREM2hi macrophages. **b**. Heatmaps displaying outgoing (Ligand) and incoming (Receptor) signalling patterns of pathways describing potential ligand-receptor interactions. Scale above heatmap indicates the relative signalling strength of a cell cluster based on all signalling pathways displayed in the heatmap. Grey bars right of the heatmap show the total signalling strength of a pathway in all cell clusters. The relative signalling strength indicated by ranging color from white (low) to green (high). All cells included in these graphs originate from the plaque.

Our data suggest that atherosclerotic plaques harbor one major CD4^+^ T cell subset that regularly undergoes antigen-specific interactions. To understand whether and how these clonally expanded T cells interact with myeloid subsets in the plaque, we selected five plaque myeloid cell populations from the overall dataset: myeloid-derived dendritic cells (DC-M), plasmacytoid dendritic cells (DC-P), proliferating macrophages (M-Prol), inflammatory macrophages (M-Inf) and foamy TREM2^hi^ macrophages (M-TREM2) (Extended Data Fig. 8a)^3^. Using CellChat, we examined potential signalling pathways between these myeloid subsets and the CD4^+^ and CD8^+^ T cells in the plaque^25^. CellChat can predict incoming (receptor) and outgoing (ligand) activity of cell signalling pathways based on scRNA-seq data, accounting for the multimeric structure of ligand–receptor complexes and the effect of co-factors on the ligand–receptor interactions. Predicted outgoing and incoming pathway signalling was displayed per cluster. Overlap between outgoing and incoming signals of a certain pathway within or between clusters indicates a possible interaction through this pathway. The different CD4^+^ T cell clusters showed different levels of relative signalling strength in the outgoing signalling patterns (top bar plot heat map, relative to outgoing signals of all pathways in the heat map), whereas CD8^+^ T cells showed little difference between the clusters (Fig. 5a and Extended Data Fig. 8b). In general, the most upregulated signalling pathway was MHCII as outgoing signal on all myeloid subsets and incoming signals in multiple CD4^+^ T cell subsets, including cluster 3 (C3). The plaque-enriched CD69^+^ C3 displayed elevated outgoing signalling patterns. Interestingly, one of the pathways that was enriched in this cluster was the CD40 pathway, involved in antigen-specific T cell activation^26^. Next, we assessed whether the CD40 pathway was also enriched as an incoming signalling pattern (Fig. 5b). Specific enrichment was observed in the M-TREM2 (foam cell) subset. Apart from the CD40 pathway, multiple other enriched pathways involved in immune synapse formation and co-stimulation could be defined between C3 and M-TREM2, including the CD99, CD6, CD40, macrophage inhibitory factor (mIF) and annexin A1 pathways (Fig. 5b)^27–30^. Together, this suggests that M-TREM2 could be involved in activation of the clonally expanded CD4^+^ T cells in atherosclerotic lesions.

### Common autoimmune phenotype in expanded plaque T cells

**Fig. 6:**
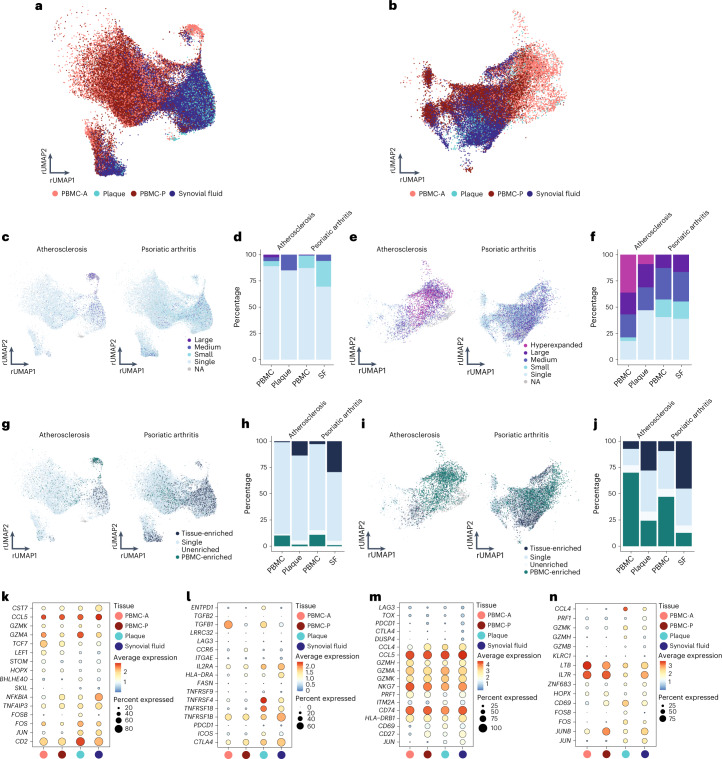
Tissue-enriched clonal expanded CD4^+^ and CD8^+^ T cells of atherosclerosis and PSA have phenotypic commonalities. **a**, Atherosclerosis and PSA CD4^+^ T cells of PBMC, plaque and SF projected on an atherosclerosis CD4^+^ T cell reference UMAP (rUMAP). **b**, Atherosclerosis and PSA CD8^+^ T cells of PBMC, plaque and SF projected on an atherosclerosis CD8^+^ T cells rUMAP. **c**, rUMAP projecting clonal expansion levels of CD4^+^ T cells in atherosclerosis and PSA. **d**, Quantification of clonal expansion levels of CD4^+^ T cells in atherosclerosis, split over PBMC and tissue. **e**, rUMAP projecting clonal expansion levels of CD8^+^ T cells in atherosclerosis and PSA. **f**, Bar plot displaying quantification of clonal expansion levels of CD8^+^ T cells in atherosclerosis, split over PBMC and tissue. **g**, rUMAP projecting tissue enrichment scores of clonotypes in CD4^+^ T cells of atherosclerosis and PSA. **h**, Bar plot with quantification of tissue enrichment scores of CD4^+^ T cells in atherosclerosis and PSA, split by PBMC and tissue. **i**, rUMAP projecting tissue enrichment scores of clonotypes in CD8^+^ T cells of atherosclerosis and PSA. **j**, Quantification of tissue enrichment scores of CD8^+^ T cells in atherosclerosis and PSA, split by PBMC and tissue. **k**–**n**, Dot plots with average expression of genes characterizing the genes underlying the overlap between atherosclerosis and PSA in CD4^+^ T_reg_ cells (C5, **k**) and T_eff_ cells (C3, **l**) and in CD8^+^ T_eff_ cells (C3, **m**; C5, **n**). Clonotype expansion levels: Single (one occurrence), Small (≤0.1%), Medium (>0.1% and ≤1%), Large (>1% and ≤10%) and Hyperexpanded (>10%), percentage of, respectively, CD4^+^ and CD8^+^ T cells. Tissue enrichment scores: Tissue-enriched (frequency expanded clone higher in tissue versus PBMC), Single (one occurrence), Unenriched (frequency expanded clone similar in PBMC versus tissue) and PBMC-enriched (frequency expanded clone higher in PBMC versus tissue).

**Extended Data Fig. 9:**
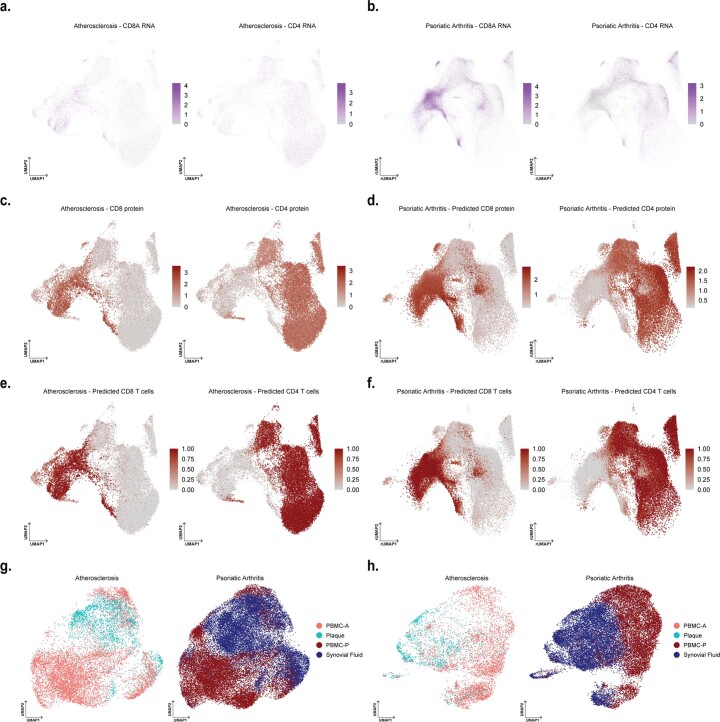
Projection of CD4^+^ and CD8^+^ T cells of integrated atherosclerosis and psoriatic arthritis single-cell TCR sequencing data on the reference UMAP projection of CD4^+^ and CD8^+^ atherosclerosis data. **a** UMAP visualization of RNA expression of CD8A and CD4 on atherosclerosis T cells. **b**. rUMAP visualization of RNA expression of CD8A and CD4 on psoriatic arthritis T cells. **c**. UMAP visualization of protein expression of CD8 and CD4 on atherosclerosis T cells. **d**. rUMAP visualization of predicted protein expression of CD8 and CD4 on psoriatic arthritis T cells. **e**. UMAP visualization of selected CD8^+^ and CD4^+^ atherosclerosis T cells. **f**. UMAP visualization of selected CD8^+^ and CD4^+^ psoriatic arthritis T cells. **g**. UMAP of integrated CD4^+^ T cells split by diseased and grouped by tissue type. **h**. UMAP of integrated CD8^+^ T cells split by diseased and grouped by tissue type.

**Extended Data Fig. 10:**
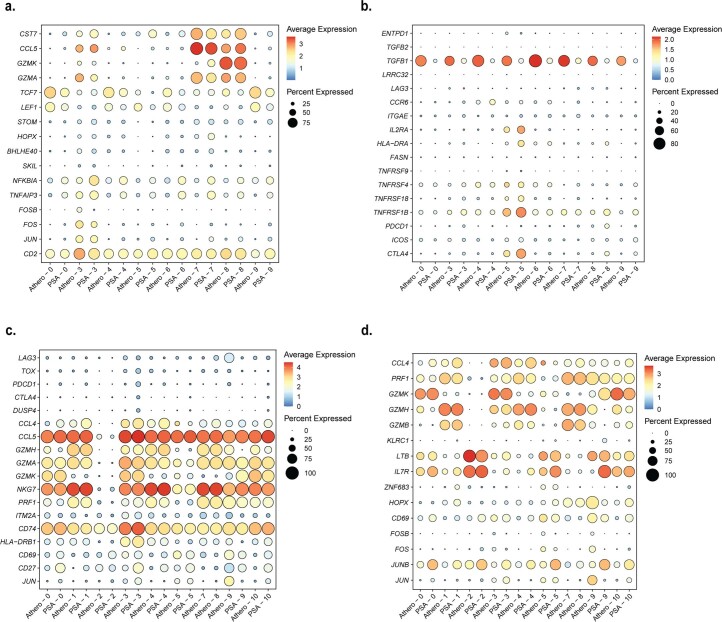
Extended dot plots with characterizing genes for atherosclerosis and psoriatic arthritis overlapping clonal expanded T cells. Dotplots with genes used to characterize overlapping clusters of atherosclerosis and psoriatic arthritis per disease and per cluster of respectively CD4^+^ cluster 3 genes (**a**), CD4^+^ cluster 5 genes (**b**) CD8^+^ cluster 3 genes (**c**) and CD8^+^ cluster 5 genes (**d**).

Based on the accumulation of plaque-enriched CD4^+^ and CD8^+^ T cell clonotypes, we hypothesized that human atherosclerosis could be characterized as an autoimmune-driven T cell response. To further confirm this hypothesis, we integrated an scTCR-seq dataset of the autoimmune disease PSA, containing data from PBMCs and synovial fluid (SF)^31^. As in this study CD45RA^−^ T cells were isolated, we excluded the naive T cell clusters from our dataset. Moreover, this study did not include feature barcoding. CD4^+^ and CD8^+^ T cells were, therefore, selected based on the labels predicted by multimodal reference mapping (Extended Data Fig. 9a–f). Subsequently, CD4^+^ and CD8^+^ T cells of both diseases were integrated (Extended Data Fig. 9g,h) and projected on the atherosclerosis CD4^+^ and CD8^+^ UMAP as reference. Remarkably, a clear overlap between PBMCs from atherosclerosis and PSA was observed in both CD4^+^ and CD8^+^ T cells. In addition, this overlap was also seen between plaque and SF for both T cell subsets (Fig. 6a,b). Next, clonal expansion levels were recalculated for both atherosclerosis and PSA (percentage of all CD4^+^ or CD8^+^ TCRs). Indeed, clonally expanded T cells were found in similar CD4^+^ and CD8^+^ T cell clusters in both diseases (Fig. 6c,e). Moreover, quantification of this clonal expansion revealed a similar distribution. An increased percentage of expanded CD8^+^ T cells versus expanded CD4^+^ T cells was detected in SF. However, as seen in atherosclerosis, the percentage of expanded CD4^+^ T cells was increased in SF compared to PBMC, whereas expanded CD8^+^ T cells did not differ between both tissues (Fig. 6d,f). Tissue enrichment scores were also determined and again displayed similarities between atherosclerosis and PSA. Tissue-enriched T cells were located in overlapping clusters in both diseases. Quantification resulted in an increase in tissue-enriched T cells in both CD4^+^ and CD8^+^ in plaque and SF compared to their matched PBMCs, although this enrichment was more prominent in SF versus plaque T cells (Fig. 6g–j). Finally, we defined the genes supporting the overlap between the atherosclerosis and PSA subsets in C3 and C5 of both CD4^+^ and CD8^+^ T cells. CD4^+^ T cells from C3 were characterized by high expression of *CCL5*, *GZMK* and *GZMA* in both plaque and SF (Fig. 6k and Extended Data Fig. 10a). Atherosclerosis-specific C3 CD4^+^ T cells had slightly increased *GZMA* expression compared to PSA PBMCs and SF. In both diseases, *FOS* and *JUN* were upregulated in tissue compared to PBMCs, whereas *FOSB* was specifically upregulated in plaque T cells. Furthermore, regulatory CD4^+^ T cells in both affected tissues appeared more active by upregulation of activation markers, including *IL2RA*, *TNFRSF4*, *TNFRSF18*, *TNFSF1B* and *CTLA4*, compared to the PBMC counterpart (Fig. 6l and Extended Data Fig. 10b). Nevertheless the T_reg_ subset showed some disparity between SF and plaque-derived cells as plaque T_reg_ also increasingly expressed *ICOS* and *ENTPD1*, compared to PSA SF-derived T_regs_. Interestingly, atherosclerosis T_reg_ cells in both PBMC and plaque had increased expression of *TGFB1* compared to the PSA T_reg_ cells. In both PSA and atherosclerosis CD8^+^ C3 T cells, expression profiles displayed a similar phenotype with high expression of T cell effector genes—for example, *CCL5*, *GZMH*, *GZMA*, *GZMK* and *NKG7* (Fig. 6m and Extended Data Fig. 10c). Lastly, CD8^+^ T cells from C5 showed upregulation of genes involved in antigen-induced TCR activation in both affected tissues (*FOS* and *JUN*) (Fig. 6n and Extended Data Fig. 10d). *FOSB* was upregulated in plaque only, similarly to CD4^+^ C3, and *JUNB* expression was increased in PSA compared to atherosclerosis. Furthermore, increased expression of *ZNF683* was observed in both diseased tissues. *GZMH* was particularly upregulated in plaque CD8^+^ T cells. To summarize, these data support the hypothesis that atherosclerosis has a considerable autoimmune component, as it has phenotypically similar clonally expanded T cells compared to the autoimmune disease PSA.

## Discussion

**Fig. 7:**
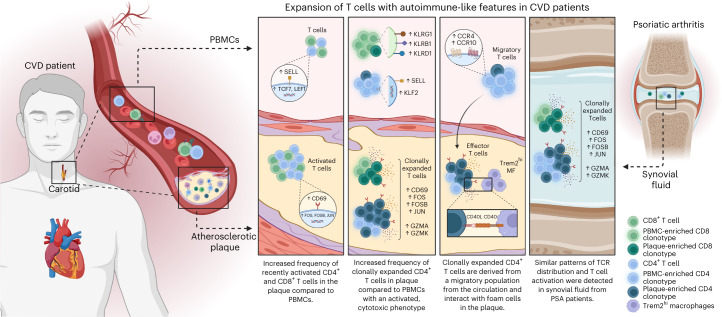
Expansion of T cells with autoimmune-like features in CVD patients. Schematic presentation of the main conclusions.

Atherosclerosis has a long history of being treated as metabolic and/or lifestyle disease, with its inflammatory component being overlooked as a potential target of intervention. Groundbreaking work earlier this century has shown that inflammation is an integral part of the disease pathophysiology, and considerable health benefits can be obtained by intervening in inflammatory cascades. Our work here takes these observations a step further and suggests that atherosclerosis is an autoimmune-like disease, with autoreactive T cells driving the inflammation process inside the plaque (Fig. 7). Classic autoimmune diseases that involve inflammation of distinct tissue, such as type I diabetes, multiple sclerosis and rheumatoid and psoriatic arthritis, are usually associated with specific HLA class II alleles, suggesting that a pathogenic CD4^+^ T cell response is a major cause of disease. Moreover, accumulation of antigen-specific T cells at the site of inflammation is a hallmark of autoimmune disease. The absence of clear associations of HLA alleles and atherosclerosis argue against the autoimmune theory in CVD^32^, yet the multifactorial nature of the disease and the large population that it affects make such associations difficult to establish. Accumulation of T cells in atherosclerotic plaques, however, is well established. Moreover, earlier studies investigating TCR diversity using TCRβ sequencing in the plaque indicated an increased clonality in the lesions compared to blood samples from patients with CVD^33^. By taking advantage of scTCR-seq here, we can combine data on distribution of TCRs with their activation state and functionality. Using this approach, we show that a selected number of effector CD4^+^ T cells and CD8^+^ T cells accumulate in the lesions and probably undergo antigen-specific activation similarly to autoimmune diseases, such as PSA. Recent work by Chowdhury et al.^10^ using a similar approach reached the same conclusion^10^; however, by using matched PBMC controls, we were able to determine that a large fraction of clonally expanded CD8^+^ T cells did not specifically accumulate in the plaque and were equally represented, or even overrepresented, in the circulation. One CD8^+^ T cell clone in particular, whose Vα TCR sequence was identified as specific for CMV, was hyperexpanded and accounted for a substantial percentage of clonally expanded T cells in the plaque while also contributing to the clonally expanded CD8^+^ T cell pool in the PBMCs of this patient. Moreover, this clone did not show a signature of recent antigen encounter. Apart from classical CD4^+^ and CD8^+^ T cells, we also identified a pro-inflammatory MAIT cell population. MAIT cells have been described in multiple autoimmune and inflammatory diseases, including PSA, with contradicting or unknown contributions to disease development. How MAIT cells contribute to atherosclerosis development and whether they are activated through their non-polymorphic MHC class I-like protein MR1 or through TCR-independent activation induced by e.g. IL-12 and IL-18 (refs. ^34–36^) needs further elucidation.

By instead focusing on the clonally enriched T cells specific for the plaque, we observed that one subset of effector CD4^+^ T cells was considerably enriched in clonally expanded TCRs and expressed genes indicative of recent antigen engagement. Although we found two such populations in the CD8^+^ T cells, their clonal enrichment was less pronounced. Interestingly, we also observed an antigen activation signature in the plaque-residing T_reg_ cells, suggesting that these T cells undergo antigen-specific interactions in the plaque. However, these T_reg_ cells did not show substantial clonal expansion, suggesting that these cells do not expand in the plaque. Instead, RNA velocity analysis suggests that T_reg_ cells are not derived from any other T cell population that we detected in PBMC or plaque. Also, we observed minimal overlapping TCR sequences between T_reg_ cells and other T cells in the plaque, in contrast to the effector CD4^+^ T cell population, which showed considerable TCR overlap with a migratory CD4^+^ T cell subset in the circulation. Previous work suggests that T_reg_ cells can lose their suppressive capacity and gain expression of pro-inflammatory markers^37^. A shift of autoreactive (ApoB100-specific) T_reg_ cells toward a T_h17_ phenotype has been associated with severity of CVD. Although the authors show in mice that this shift happens independent of the TCR clonotypes, our data argue against such a shift and suggest that T_reg_ cells and effector CD4^+^ T cells do not derive from the same ancestor but, rather, develop independent of one another. Alternatively, the number of TCRs detected here may not have been sufficient to find overlapping sequences between T_reg_ cells and effector CD4^+^ T cells. Also, it is unknown whether ApoB100-specific T cells undergo antigen-specific interaction in the plaque, and, because the antigen specificity of T cells investigated in this study are unknown, it is possible that we did not examine ApoB100-specific CD4^+^ and CD8^+^ T cells here.

We attempted to cluster the TCRs in silico using GLIPH2 and GIANA algorithms^38,39^, which are based on CDR3β similarity, as this is proposed to be an attractive way to cluster TCRs for a specific antigen together. However, a convincing clustering of plaque-enriched clonotypes was not observed in our dataset. The current clustering algorithms may have some limitations, which, in our data, was illustrated by co-clustering of CD4^+^ T cell-derived and CD8^+^ T cell-derived clonotypes, which was resolved only if the CDR3α sequence was included. Moreover, we observed diffuse clustering of clonotypes previously reported as ApoB100 specific^40^, suggesting that the current algorithms are not specific enough to resolve TCR clustering in atherosclerosis. Therefore, we think that a more stringent approach that includes both CDR3α and CDR3β needs to be developed.

As we observe antigen-specific activation in both the effector and T_reg_ subsets, it is currently unclear what the overall effect of TCR engagement in the lesion is. Previous work in mice has shown mixed results with MHCII^−/−^apoE^−/−^ mice, suggesting that this interaction is protective, whereas various papers suggest a pathogenic role for CD4^+^ T cells in atherosclerosis^41,42^. Interestingly, our work identifies several pathways involved in co-stimulation and immunological synapse formation that potentially drive pathogenic interactions of effector CD4^+^ T cells with the M-TREM2 (foam cell) population. When limited to effector CD4^+^ T cell populations, these may be specific and druggable targets. For instance, the expression of *CD40LG* on the clonally enriched effector population suggests active signalling to foam cells through CD40. This co-stimulatory pathway and that of other TNF superfamily member has been extensively studied in mouse models of atherosclerosis and is the subject of a clinical study^43,44^. The observation of antigen-specific T_reg_ interaction also provides a rationale for potential therapeutic possibilities, such as expanding these cells by means of vaccination or development of tolerogenic chimeric antigen receptor (CAR) T cells. Identification of the antigen(s) driving T_reg_ interaction in the plaque will be crucial for this development. Potential antigens, such ApoB100, heat shock proteins and fibronectin, have been suggested as potential self-antigens and have shown therapeutic potential as antigens in mouse models^45–47^ and may serve as a potential starting point for vaccine development. Thus, here we highlight an autoimmune component to the pathophysiology of atherosclerosis, and we confirm a rationale for immunotherapeutic interventions in CVD.

## Methods

### Patient cohorts

For flow cytometry (cohort 1) and bulk TCRβ sequencing (cohort 3), whole blood and atherosclerotic plaques were obtained from, respectively, 61 and 10 patients who underwent carotid endarterectomy (CEA) surgery at the Haaglanden Medical Center Westeinde (HMC; The Hague, The Netherlands). The study was approved by the Medical Ethics Committee of the HMC (study approval number, cohort 1: 17-046, protocol number NL57482.098.17; study approval number, cohort 3: Z19.075, protocol number NL71516.058.19). For scTCR-seq, whole blood and atherosclerotic plaques were obtained from three male patients who underwent CEA (cohort 2). Patients were included in the Athero-Express biobank (www.atheroexpress.nl), an ongoing biobank study at the University Medical Centre Utrecht (UMCU)^48^. The study was approved by the Medical Ethics Committee of the UMCU (study approval number: TME/C-01.18, protocol number 03/114). All blood samples were collected by venipuncture before surgery. Atherosclerosis specimens were obtained from primary CEAs, and estenotic plaques were excluded due to their different plaque composition as compared to primary atherosclerotic plaques^49^. Informed consent was obtained from all patients involved in this study.

### Whole blood processing

Peripheral venous blood was collected in K2-EDTA blood tubes (BD Vacutainer). For scTCR-seq, blood was processed within 10 minutes after withdrawal (cohort 2). For both cohort 1 and cohort 2, blood was diluted 1:2 in PBS containing 2% FCS. A density gradient was created using SepMate PBMC isolation tubes (STEMCELL Technologies) containing Ficoll-Paque Premium (GE Healthcare). Cells were centrifuged at 1,200*g* for 10 minutes at room temperature. The intermediate layer containing PBMCs was isolated and washed twice with PBS + 2% FCS (250*g*, 10 minutes, room temperature). Cells were taken up in PBS + 1% BSA until further processing. For cohort 3, whole blood samples were lysed twice with ACK lysis buffer in PBS (1:10) for 10 minutes at room temperature and washed with PBS (300*g*, 5 minutes). Cells were taken up in RPMI + 1% FCS and cryostored in CryoStor cell cryopreservation medium (Sigma-Aldrich) until further use.

### Human atherosclerotic plaque cell isolation

Human carotid plaques were collected during CEA; the culprit segment (5 mm) was used for histology and embedded in paraffin as described elsewhere^48^. In brief, culprit segments were fixed in 4% formaldehyde and decalcified in 10% EDTA, pH 7.5. Afterwards, culprit segments were embedded in paraffin. Time between surgical removal and plaque processing did not exceed 10 minutes. The inclusion of a small medial layer in the dissected tissue could not be excluded during the surgical procedure. The remainder of the plaque was washed in RPMI and minced into small pieces with a razor blade. The tissue was then digested in RPMI 1640 containing 2.5 mg ml^−1^ of collagenase IV (Thermo Fisher Scientific), 0.25 mg ml^−1^ of DNAse I (Sigma-Aldrich) and 2.5 mg ml^−1^ of Human Albumin Fraction V (MP Biomedicals) at 37 °C for 30 minutes. In cohort 2, 1 µM flavopiridol (Selleck Chemicals) was added to the digestion mixture. Subsequently, the plaque cell suspension was filtered through a 70-µm cell strainer and washed with RPMI 1640. Cells were kept in RPMI 1640 with 1% FCS until subsequent staining for flow cytometry (cohort 1), feature barcoding and FACS (cohort 2) or cryostored in CryoStor cell cryopreservation medium (Sigma-Aldrich) until further use.

### Flow cytometry

Single-cell suspensions from blood and plaque from cohort 1 were stained with a mixture of extracellular antibodies for 30 minutes at 37 °C (Supplementary Table 4). All measurements were performed on a CytoFLEX S (Beckman Coulter) and analysed with FlowJo version 10.7 (Tree Star). A Shapiro log-normality test was performed, and a two-tailed Mann–Whitney test was performed using GraphPad analysis software to determine significance.

### Antibody staining for feature barcoding and FACS

#### PBMC

PBMCs of cohort 2 were stained with TotalSeq-C antibodies against CD3, CD4, CD8 and CD14 (Supplementary Table 4). Antibody pools containing 0.25 µg per antibody were prepared in labeling buffer (PBS + 1% BSA) and spun down at 14,000*g* for 10 minutes at room temperature, and supernatant was collected for further staining. First, cells were stained with Human TruStain FcX (BioLegend) for 10 minutes at 4 °C. Next, the antibody pool supernatant was added and incubated for 30 minutes at 4 °C. Cells were washed three times with labeling buffer at 400*g* for 5 minutes at 4 °C. Next, cells were taken up in PBS + 0.4% BSA and further processed with 10x Genomics.

#### Plaque

Single-cell suspensions of plaques of cohort 2 were stained with TotalSeq-C antibodies against CD3, CD4, CD8 and CD14 (Supplementary Table 4). Antibody pools containing 0.25 µg per antibody and plaque (1 µg per antibody) single-cell suspensions were prepared in labeling buffer (PBS + 1% BSA) and spun down at 14,000*g* for 10 minutes at room temperature, and supernatant was collected for further staining. First, cells were stained with Human TruStain FcX (BioLegend) for 10 minutes at 4 °C. Next, the antibody pool supernatant was added together with Calcein AM (1:1,000, Thermo Fisher Scientific), Hoechst (1:1,000, Thermo Fisher Scientific) and CD45-PECy7 (1:200, clone HI30, BD Biosciences) and incubated for 30 minutes at 4 °C. Cells were washed three times with labeling buffer at 400*g* for 5 minutes at 4 °C. Next, cells were taken up in PBS + 2% FBS. Live CD45^+^ plaque cells were sorted using the BD FACSAria II (BD Biosciences) in PBS + 0.04% BSA and further processed with 10x Genomics.

### scTCR-seq by 10x Genomics

scTCR-seq was performed on PBMCs and live CD45^+^ plaque cell suspensions from cohort 2 using 10x Genomics 5′ Single Cell Immune Profiling technology. Sequencing libraries were prepared using the 5′ version 1.1 chemistry following standard 10x Genomics protocol. Sequencing was performed using the Illumina NovaSeq 6000 (Novogene).

### Bulk TCRβ sequencing

Genomic DNA was extracted from plaque single-cell suspensions and matched PBMC samples (cohort 3) using a DNA extraction kit in accordance with the manufacturer’s instructions (Qiagen). Sequencing of the VDJ locus was performed using the Adaptive Biotechnologies TCRβ sequencing platform.

### scTCR-seq data processing, clustering and clonotype quantification

scTCR-seq data analyses were executed in R-4.0.1 and R-4.1.3 environments, primarily using Seurat (version 4.0.0–4.1.1)^50,51^. scTCR-seq data were processed as previously described^51,52^. In short, reads were filtered for mitochondrial, ribosomal genes and long non-coding RNA genes. To remove apoptotic cells, low-quality cells and doublets, only cells with a gene expression below 2% for KCNQ1OT1, below 2% for UGDH-AS1, below 2% for GHET1 and expressing between 200 and 5,000 genes were used for further analysis. Quality control (QC)-filtered PBMC and plaque Seurat objects were first merged per patient, after which the patient-merged Seurat objects were normalized using the SCT method, integrated using rpca reduction and clustered according to the Seurat ‘scRNA-seq integration’ vignette. VDJ sequencing data were imported into Seurat using the combineExpression function of scRepertoire (version 1.4.0)^53^. The complete integrated dataset was mapped to the pbmc_multimodal.h5seurat dataset (https://atlas.fredhutch.org/data/nygc/multimodal/pbmc_multimodal.h5seurat) to transfer cell type labels to the integrated Seurat object.

For subclustering, T cells were selected from the complete integrated dataset, taking the clusters with protein expression of CD3, CD4 and CD8 and without CD14 expression (ADT assay). Before reclustering the T cells, variable TCR genes were removed from the variable genes list, before principal component analysis (PCA) and clustering, to avoid clustering based on TCR, interfering with clustering on T cell phenotypes. However, TCR genes were not removed from the dataset. Separate CD4^+^ T cell and CD8^+^ T cell objects were then created by subsetting the T cell object based on, respectively, protein expression of CD4 > 0.75 and CD8 > 1.0 in the ADT assay. Custom clonotype counting functions were used to quantify the clonotype content of the individual samples based on the amino acid sequences of the TCRs. Clonotype frequencies relating to the total TCR repertoire per patient, per tissue are depicted in the atherosclerosis figures. Volcano plots were created using EnhancedVolcano (version 1.8.0)^54^. For all volcano plots, the FindMarkers function of Seurat was used to define differential genes between both groups by using a non-parametric Wilcoxon rank-sum test to determine significance. To assess the differentiation trajectories of the CD4^+^ T cells and CD8^+^ T cells, Monocle3 and velocyto.R (version 0.6) were used^22,55^. To assess possible interactions of antigen-presenting cells and T cells in the plaque, CellChat (version 1.4.0) was used^25^.

### Definition of clonotype expansion levels and tissue enrichment scores

The TCR amino acid sequences were used to define the clonotypes. The clonotype abundance of a clonotype was calculated as the percentage of cells expressing a certain clonotype within a tissue of a patient, divided by the total number of cells in which a TCR was detected in the same tissue of the same patient. Based on the number and percentage of cells expressing the same clonotype, clonotypes were classified as Hyperexpanded, Large, Medium, Small or Single in the tissues of the patients (Supplementary Table 5). Furthermore, the tissue enrichment of clonotypes was determined according to the parameters listed in Supplementary Table 6.

### Integration with PSA scTCR-seq data

T cells from our scTCR-seq atherosclerosis dataset were compared with TCR-seq data from donor-matched PBMCs and synovial tissue from patients with PSA (ArrayExpress: E-MTAB-9492; European Genome-phenome Archive: EGAS00001002104)^31^. The same QC and processing steps were performed for the PSA dataset as described above for our atherosclerosis dataset. Subsequently, the integrated PSA dataset was mapped to the UMAP reduction of our complete T cell object, using our atherosclerosis dataset as reference. Because CD4^+^ T cells and CD8^+^ T cells could not be separated cleanly based on the clustering, and the PSA dataset does not contain protein expression data, the atherosclerosis dataset and the PSA dataset were divided based on the predicted cell type (CD4 T cell or CD8 T cell), derived from the pbmc_multimodal.h5seurat dataset. Subsequently, the atherosclerosis and PSA CD4^+^ T cell and CD8^+^ T cell datasets were split by patient and reintegrated as previously described for the atherosclerosis object, to form a CD4^+^ T cell object and a CD8^+^ T cell object containing atherosclerosis-derived and PSA-derived T cells. Then, the integrated datasets were mapped to our original CD4^+^ T cell and CD8^+^ T cell UMAP reductions. Because the PSA dataset is devoid of naive T cells due to the T cell isolation procedure used by Penkava et al.^31^, naive T cell clusters were removed from the CD4^+^ T cell dataset (clusters 1 and 2) and the CD8^+^ T cell dataset (cluster 6) before quantification of the clonotype abundance^31^.

### Reporting summary

Further information on research design is available in the Nature Portfolio Reporting Summary linked to this article.

### Supplementary information

Reporting Summary

Supplementary Tables 1 and 4–6. Table 1: Baseline characteristics of patient cohorts. Table 4: Extracellular and intracellular antibodies used for flow cytometry and feature barcoding. Table 5: Clonal expansion levels. Table 6: Tissue enrichment scores.

Supplementary Table 2. Differentially expressed genes of T cell, CD4^+^ and CD8^+^ T cell clusters.

Supplementary Table 3. Per-patient display of clonal expansion levels and tissue enrichment scores for the whole dataset.

### Source data

Source Data Fig. 1 Statistical Source Data

Source Data Fig. 2 Statistical Source Data

Source Data Extended Data Fig. 3 Statistical Source Data

Source Data Extended Data Fig. 6 Statistical Source Data

Source Data Extended Data Fig. 7 Statistical Source Data

## Data Availability

The raw scTCR-seq data from the Athero-Express cohort are not publicly available due to research participant privacy/consent. These data and the bulk TCRβ sequencing data can be accessed via DataverseNL at this address: 10.34894/DDYKLL. There are restrictions on use by commercial parties and on sharing openly based on (inter)national laws and regulations and written informed consent. Therefore, these data (and additional clinical data) are available only upon discussion and signing a data sharing agreement (see Terms of Access in DataverseNL) and within a specially designed UMCU-provided environment. Open-source scTCR-seq data from donor-matched PBMCs and synovial tissue from patients with PSA that we used in this study are publicly available (ArrayExpress: E-MTAB-9492; European Genome-phenome Archive: EGAS00001002104)^31^.

## Code availability

In silico data analysis was performed using custom-made R scripts designed specifically for this study and/or based on the recommended pipelines from pre-existing packages listed above. R scripts are available via Zenodo (10.5281/zenodo.7415207).
