# Supplementary material for: Single-cell T cell receptor sequencing of paired human atherosclerotic plaques and blood reveals autoimmune-like features of expanded effector T cells
Source: Nat Cardiovasc Res. 2023 Jan 30;2(2):112–25. doi: 10.1038/s44161-022-00208-4 (PMC11041750; doi:10.1038/s44161-022-00208-4)
Supplement: Supplementary file 1 — Reporting Summary [file 44161_2022_208_MOESM1_ESM.pdf]

nature portfolio

Corresponding author(s): Bram Slütter and Ilze Bot

Last updated by author(s): Dec 8, 2022

Reporting Summary

Nature Portfolio wishes to improve the reproducibility of the work that we publish. This form provides structure for consistency and transparency in reporting. For further information on Nature Portfolio policies, see our Editorial Policies and the Editorial Policy Checklist.

Statistics

For all statistical analyses, confirm that the following items are present in the figure legend, table legend, main text, or Methods section.

n/a	Confirmed	
☐	☒	The exact sample size (*n*) for each experimental group/condition, given as a discrete number and unit of measurement
☐	☒	A statement on whether measurements were taken from distinct samples or whether the same sample was measured repeatedly
☐	☒	The statistical test(s) used AND whether they are one- or two-sided<br>*Only common tests should be described solely by name; describe more complex techniques in the Methods section.*
☐	☒	A description of all covariates tested
☐	☒	A description of any assumptions or corrections, such as tests of normality and adjustment for multiple comparisons
☐	☒	A full description of the statistical parameters including central tendency (e.g. means) or other basic estimates (e.g. regression coefficient) AND variation (e.g. standard deviation) or associated estimates of uncertainty (e.g. confidence intervals)
☐	☒	For null hypothesis testing, the test statistic (e.g. *F*, *t*, *r*) with confidence intervals, effect sizes, degrees of freedom and *P* value noted<br>*Give P values as exact values whenever suitable.*
☒	☐	For Bayesian analysis, information on the choice of priors and Markov chain Monte Carlo settings
☒	☐	For hierarchical and complex designs, identification of the appropriate level for tests and full reporting of outcomes
☒	☐	Estimates of effect sizes (e.g. Cohen's *d*, Pearson's *r*), indicating how they were calculated

Our web collection on statistics for biologists contains articles on many of the points above.

Software and code

Policy information about availability of computer code

Data collection	We used CellRanger (v6.0.2, www.10xgenomics.com) to demultiplex the samples and assemble the VDJ data to the cells. Gene expression data was referenced to ENSEMBL GRCh38-2020-A and VDJ sequences were referenced to ENSEMBL GRCh38 v3.1.0.
Data analysis	Single-cell TCR sequencing data analyses were executed in R-4.0.1 and R-4.1.3 environments. Data analysis was mainly performed using Seurat (version 4.0.0 – 4.1.1) and several pre-existed coding packages (scRepertoire v1.4.0, EnhancedVolcano v1.8.0, Monocle v3, velocyto.R v0.6, CellChat v1.4.0). All other analysis were performed using custom made R scripts designed specifically for this study. Custom R scripts are availabe via Zenodo (https://doi.org/10.5281/zenodo.7415207).

For manuscripts utilizing custom algorithms or software that are central to the research but not yet described in published literature, software must be made available to editors and reviewers. We strongly encourage code deposition in a community repository (e.g. GitHub). See the Nature Portfolio guidelines for submitting code & software for further information.

Data

Policy information about availability of data

All manuscripts must include a data availability statement. This statement should provide the following information, where applicable:

- Accession codes, unique identifiers, or web links for publicly available datasets
- A description of any restrictions on data availability
- For clinical datasets or third party data, please ensure that the statement adheres to our policy

Bulk TCRb sequencing data and the raw data files of the single-cell TCR sequencing experiments are available in a DataVerseNL repository (https://doi.org/10.34894/DDYKLL). Open source scTCRseq data from donor-matched PBMC and Synovial Tissue from Psoriatic arthritis (PSA) patients (ArrayExpress: E-MTAB-9492, European Genome-phenome Archive: EGAS00001002104) were used in this study.

Human research participants

Policy information about studies involving human research participants and Sex and Gender in Research.

Reporting on sex and gender	We refer to biological sex and report on sex differences when relevant throughout the manuscript.
Population characteristics	Supplemental Table 1 lists all relevant covariates of the patient cohorts used in this study. Cohort 1 consisted of 61 patients with a mean age of 74 and 67% male sex. Cohort 2 consisted of 3 pateients with a mean age of 75 and 100% male sex. Cohort 3 consisted of 10 patients with a mean age of 77 and 60% male sex.
Recruitment	Participants are recruited by the responsible medical doctor prior to the carotid endarterectomy surgery. Patients that are younger than 18 or that are not able to give consent are excluded from the study. Included patients will get a study number and will remain anonymous for the rest of the study. Patients were selected on availability only.
Ethics oversight	Cohort 1 and cohort 3 have been approved by the Medical Ethics Committee of the HMC (Study approval number Cohort 1: 17-046, protocol number NL57482.098.17 and Cohort 3: Z19.075, protocol number NL71516.058.19). Cohort 2 has been approved by the Medical Ethics Committee of the UMCU (Study approval number: TME/C-01.18, protocol number 03/114)

Note that full information on the approval of the study protocol must also be provided in the manuscript.

Field-specific reporting

Please select the one below that is the best fit for your research. If you are not sure, read the appropriate sections before making your selection.

☒ Life sciences ☐ Behavioural & social sciences ☐ Ecological, evolutionary & environmental sciences

For a reference copy of the document with all sections, see nature.com/documents/nr-reporting-summary-flat.pdf

Life sciences study design

All studies must disclose on these points even when the disclosure is negative.

Sample size	No a priori sample size calculations have been performed. But we have included all possible material that was available at the time of the experiments.
Data exclusions	For flow cytometry in figure 1, two samples have been omitted due to lack of measured events. No other data points have been excluded.
Replication	Bulk TCRbeta sequencing has been performed to verify the single-cell TCR sequencing. single-cell TCR sequencing data shows little interpatient variability (Extended Data Figure 2e-f).
Randomization	Randomization is not applicable.
Blinding	Blinding is not applicable.

Reporting for specific materials, systems and methods

We require information from authors about some types of materials, experimental systems and methods used in many studies. Here, indicate whether each material, system or method listed is relevant to your study. If you are not sure if a list item applies to your research, read the appropriate section before selecting a response.

Materials & experimental systems

n/a	Involved in the study
☐	☒ Antibodies
☒	☐ Eukaryotic cell lines
☒	☐ Palaeontology and archaeology
☒	☐ Animals and other organisms
☒	☐ Clinical data
☒	☐ Dual use research of concern

Methods

n/a	Involved in the study
☒	☐ ChIP-seq
☐	☒ Flow cytometry
☒	☐ MRI-based neuroimaging

Antibodies

Antibodies used

All antibodies used are listed in supplemental table 4 and in the list below:

TotalSeq-C:
CD3-C0034: Clone: UCHT1; Cat.No.: 300479; Company: Biolegend
CD4-C0072: Clone: RPA-T4; Cat.No.: 300567; Company: Biolegend
CD8-C0080: Clone: RPA-T8; Cat.No.: 301071; Company: Biolegend
CD14-C0051: Clone: 63D3; Cat.No. 367137; Company: Biolegend

Flow Cytometry
CD45-APC: Clone 2D1; Cat.No. 368512; Company: Biolegend
CD3-PercP-Cy5.5: Clone: OKT3; Cat.No.: 317335; Company: Biolegend
CD4-BV650: Clone: RPA-T4; Cat.No. 300536; Company: Biolegend
CD8-PE-Cy7: Clone: SK1; Cat.No.: 344711; Company: Biolegend
CD69-BV510: Clone: FN50; Cat.No.: 310936; Company: Biolegend
Fixable Viability Dye-eFluor 780: Cat.No.: 65-0865-18; Company: eBioscience
TruStain FcX: Cat.No.: 422302; Company: Biolegend

Fluorescent Activated Cell Sorting:
Calcein AM: Cat.No. C1430; Company: ThermoFisher
Hoechst 33342: Cat. No. 62249
CD45-PE-Cy7: Clone: HI30; Cat.No: 557738; Company: BD Biosciences

Validation

All antibodies underwent significant quality control by the manufacturer. As stated on Biolegend's website (https://www.biolegend.com/en-us/quality/quality-control), specificity testing for flow cytometry antibodies has been performed on 1-3 target cell types with either single- or multi-color analysis (including positive and negative cell types). Once specificity is confirmed, each new lot must perform with similar intensity to the in-date reference lot. Brightness (MFI) is evaluated from both positive and negative populations. Each lot product is validated by QC testing with a series of titration dilutions. We have used their suggested dilutions for flow cytometry staining. For TotalSeq Antibodies, Bulk lots are tested by PCR and sequencing to confirm the oligonucleotide barcodes. They are also tested by flow cytometry to ensure the antibodies recognize the proper cell populations. Bottled lots are tested by PCR and sequencing to confirm the oligonucleotide barcodes. We have tested the used dilution for our TotalSeq-C antibodies with flow cytometry.

Flow Cytometry

Plots

Confirm that:

☒ The axis labels state the marker and fluorochrome used (e.g. CD4-FITC).

☒ The axis scales are clearly visible. Include numbers along axes only for bottom left plot of group (a 'group' is an analysis of identical markers).

☒ All plots are contour plots with outliers or pseudocolor plots.

☒ A numerical value for number of cells or percentage (with statistics) is provided.

Methodology

Sample preparation

Whole blood processing
Peripheral venous blood was collected in K2-EDTA blood tubes (BD Vacutainer). For single-cell TCR sequencing, blood was processed within 10 minutes after withdrawal (Cohort 2). For both Cohort 1 and 2, blood was diluted 1:2 in Phosphate Buffered Saline (PBS) containing 2% Fetal Calf Serum (FCS). A density gradient was created using SepMate™ PBMC isolation tubes (STEMCELL Technologies) containing Ficoll-Paque Premium™ (GE Healthcare). Cells were centrifuged at 1200xg for 10 minutes at room temperature. The intermediate layer containing peripheral blood mononuclear cells (PBMC) was isolated and washed twice with PBS + 2% FCS (250xg, 10 minutes, room temperature). Cells were taken up in PBS + 1% Bovine Serum Albumin (BSA) until further processing. For Cohort 3, whole blood samples were lysed twice with ACK lysis buffer in PBS(1:10) for 10 minutes at RT and washed with PBS (300xg, 5 minutes). Cells were taken up in RPMI + 1% FCS and cryostored in Cryostor cell cryopreservation medium (Sigma-Aldrich) until further use.

Human atherosclerotic plaque cell isolation
Human carotid plaques were collected during CEA; the culprit segment (5 mm) was used for histology and embedded in paraffin as described elsewhere.45 In brief, culprit segments were fixed in 4% formaldehyde and decalcified in 10% EDTA pH 7.5. Afterwards, culprit segments were embedded in paraffin. Time between surgical removal and plaque processing did not exceed 10 minutes. The inclusion of a small medial layer in the dissected tissue could not be excluded during the surgical procedure. The remainder of the plaque washed in RPMI and minced into small pieces with a razor blade. The tissue was then digested in RPMI 1640 containing 2.5 mg/mL Collagenase IV (ThermoFisher Scientific), 0.25 mg/mL DNAse I (Sigma), 2.5 mg/mL Human Albumin Fraction V (MP Biomedicals) at 37oC for 30 minutes. In Cohort 2, 1 µM Flavopiridol (Selleckchem) was added to the digestion mixture. Subsequently, the plaque cell suspension was filtered through a 70 µm cell strainer and washed with RPMI 1640. Cells were kept in RPMI 1640 with 1% Fetal Calf Serum until subsequent staining for flow cytometry (Cohort 1), Feature Barcoding and fluorescence-activated cell sorting (Cohort 2) or cryostored in Cryostor cell cryopreservation medium (Sigma-Aldrich) until further use.

Instrument	Cytoflex S (Beckman Coulter) and BD FACS Aria II (BD Biosciences)
Software	FlowJo v10.7 (Treestar, San Carlos, CA, USA) and BD FACSDiva software (BD Biosciences)
Cell population abundance	Purity of post flowsort fractions was not further investigated as scRNAseq allows identification of all relevant (CD45+) populations regardless of the purity.
Gating strategy	Gating strategy Figure 1: Single cells were gated, Live CD45+ cells were selected, for plaque cells an extra lymphocyte gate was placed based on FSC-AxSSC-A. Subsequently CD3+ cells were selected. Within the CD3+ cells, both CD4+ and CD8+ T cells were gated and in both populations CD69+ cells were gated (See Extended Data Fig.1)<br>Gating Strategy FACS: Single cells were gated. Live cells (Calcein AM+ Hoechst+) were selected. Within the live cell gate, CD45 + cells were gated and selected for sorting (See Extended Data Fig.2).

☒ Tick this box to confirm that a figure exemplifying the gating strategy is provided in the Supplementary Information.
